# Evaluative Methodology for HRD Testing: Development of Standard Tools for Consistency Assessment

**DOI:** 10.1093/gpbjnl/qzaf017

**Published:** 2025-02-27

**Authors:** Zheng Jia, Yaqing Liu, Shoufang Qu, Wenbin Li, Lin Gao, Lin Dong, Yun Xing, Yadi Cheng, Huan Fang, Yuting Yi, Yuxing Chu, Chao Zhang, Yanming Xie, Chunli Wang, Zhe Li, Zhihong Zhang, Zhipeng Xu, Yang Wang, Wenxin Zhang, Xiaoping Gu, Shuang Yang, Jinghua Li, Liangshen Wei, Yuanting Zheng, Guohui Ding, Leming Shi, Xin Yi, Jianming Ying, Jie Huang

**Affiliations:** Department of In Vitro Diagnostic Reagent, National Institutes for Food and Drug Control (NIFDC), Beijing 100050, China; State Key Laboratory of Genetic Engineering, School of Life Sciences, Human Phenome Institute, and Shanghai Cancer Center, Fudan University, Shanghai 200438, China; Department of In Vitro Diagnostic Reagent, National Institutes for Food and Drug Control (NIFDC), Beijing 100050, China; Department of Pathology, State Key Laboratory of Molecular Oncology, National Cancer Center/National Clinical Research Center for Cancer/Cancer Hospital, Chinese Academy of Medical Sciences and Peking Union Medical College, Beijing 100021, China; GenePlus-Shenzhen Institute, Shenzhen 518000, China; Department of Pathology, State Key Laboratory of Molecular Oncology, National Cancer Center/National Clinical Research Center for Cancer/Cancer Hospital, Chinese Academy of Medical Sciences and Peking Union Medical College, Beijing 100021, China; GenePlus-Beijing Institute, Beijing 102200, China; GenePlus-Beijing Institute, Beijing 102200, China; GenePlus-Beijing Institute, Beijing 102200, China; GenePlus-Beijing Institute, Beijing 102200, China; GenePlus-Beijing Institute, Beijing 102200, China; GenePlus-Shenzhen Institute, Shenzhen 518000, China; State Key Laboratory of Genetic Engineering, School of Life Sciences, Human Phenome Institute, and Shanghai Cancer Center, Fudan University, Shanghai 200438, China; Technology Development Center-Bioinformatics Group, BGI Genomics, Shenzhen 518081, China; Clin Lab, BGI Genomics, Tianjin 300308, China; Precision Scientific (Beijing) Co., Ltd., Beijing 100085, China; Burning Rock Biotech, Guangzhou 510300, China; The International Human Phenome Institutes (Shanghai), Shanghai 200082, China; Department of In Vitro Diagnostic Reagent, National Institutes for Food and Drug Control (NIFDC), Beijing 100050, China; Department of In Vitro Diagnostic Reagent, National Institutes for Food and Drug Control (NIFDC), Beijing 100050, China; Amoy Diagnostics Co., Ltd., Xiamen 361027, China; Amoy Diagnostics Co., Ltd., Xiamen 361027, China; GeneWell Biotechnology Co., Ltd., Shenzhen 518081, China; GeneWell Biotechnology Co., Ltd., Shenzhen 518081, China; State Key Laboratory of Genetic Engineering, School of Life Sciences, Human Phenome Institute, and Shanghai Cancer Center, Fudan University, Shanghai 200438, China; The International Human Phenome Institutes (Shanghai), Shanghai 200082, China; State Key Laboratory of Genetic Engineering, School of Life Sciences, Human Phenome Institute, and Shanghai Cancer Center, Fudan University, Shanghai 200438, China; The International Human Phenome Institutes (Shanghai), Shanghai 200082, China; GenePlus-Beijing Institute, Beijing 102200, China; Department of Pathology, State Key Laboratory of Molecular Oncology, National Cancer Center/National Clinical Research Center for Cancer/Cancer Hospital, Chinese Academy of Medical Sciences and Peking Union Medical College, Beijing 100021, China; Department of In Vitro Diagnostic Reagent, National Institutes for Food and Drug Control (NIFDC), Beijing 100050, China

**Keywords:** Homologous recombination deficiency, Reference material, Reference dataset, Performance evaluation, Precision medicine

## Abstract

Homologous recombination deficiency (HRD) has emerged as a critical prognostic and predictive biomarker in oncology. However, current testing methods, especially those reliant on targeted panels, are plagued by inconsistent results from the same samples. This highlights the urgent need for standardized benchmarks to evaluate HRD assay performance. In phases IIa and IIb of the Chinese HRD Harmonization Project, we developed ten pairs of well-characterized DNA reference materials derived from lung, breast, and melanoma cancer cell lines and their matched normal cell lines, keeping each paired with seven cancer-to-normal mass ratios. Reference datasets for allele-specific copy number variations (ASCNVs) and HRD scores were established and validated using three sequencing methods and nine analytical pipelines. The genomic instability scores (GISs) of the reference materials ranged from 11 to 96, enabling validation across various thresholds. The ASCNV reference datasets covered a genomic span of 2340 to 2749 Mb, equivalent to 81.2% to 95.4% of the autosomes in the 37d5 reference genome. These benchmarks were subsequently utilized to assess the accuracy and reproducibility of four HRD panel assays, revealing significant variability in both ASCNV detection and HRD scores. The concordance between panel-detected GISs and reference GISs ranged from 0.81 to 0.94, with only two assays exhibiting high overall agreement with Myriad MyChoice CDx for HRD classification. This study also identified specific challenges in ASCNV detection in HRD-related regions and the profound impact of high ploidy on consistency. The established HRD reference materials and datasets provide a robust toolkit for objective evaluation of HRD testing.

## Graphical Abstract

**Figure:**
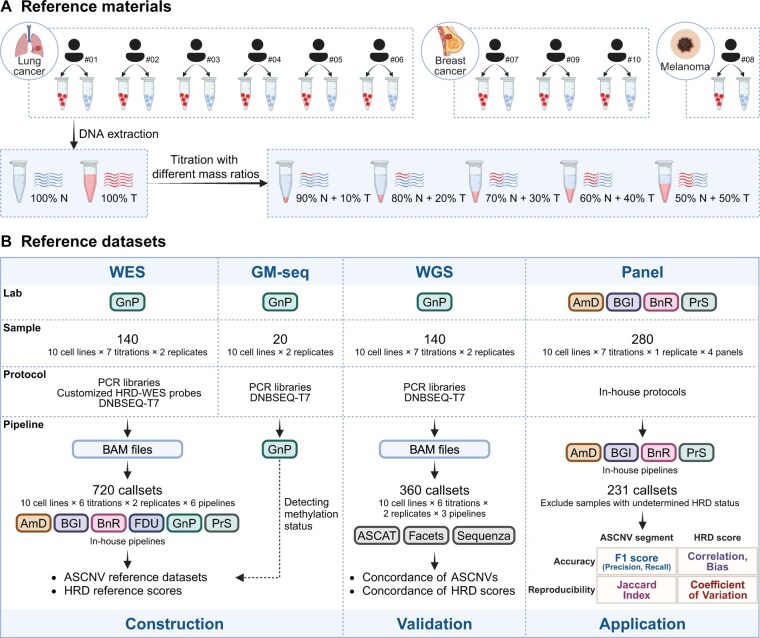

## Introduction

Homologous recombination deficiency (HRD) signifies a cellular impediment in rectifying double-stranded breaks through the homologous recombination repair (HRR) pathway, resulting in compromised DNA repair, genomic instability, and tumorigenesis [1−3]. In patients with HRD, the application of poly (ADP-ribose) polymerase (PARP) inhibitors (PARPis) disrupts the PARP-mediated single-stranded DNA damage repair pathway, resulting in the accumulation of double-stranded breaks. This, in turn, catalyzes apoptosis in tumor cells, characterized as “synthetic lethal” [4–6]. Moreover, HRR is the most precise and high-fidelity system for repairing DNA inter-strand crosslinks, suggesting that tumor cells with HRD will be sensitive to platinum-based chemotherapeutic agents that induce DNA crosslinks [7,8]. Consequently, HRD is considered a promising pan-cancer biomarker, and has been used to guide the implementation of PARPis and platinum-based chemotherapeutic strategies in ovarian [9], breast [10], endometrial [11], pancreatic ductal [12], and prostate [13,14] cancers.

A diverse spectrum of DNA-based HRD detection methods has emerged, encompassing genetic tests targeting DNA damage response (DDR) or HRR genes, and genomic tests characterizing “genomic scars” [15,16]. The former strategy is to identify “causes”, *e.g.*, cancer-susceptibility genes such as *BRCA1/2*, potentially overlooking alternative mechanisms contributing to HRD, including epigenetic modifications [17]. The latter strategy assesses “consequences” rather than “causes”, involving mutation signatures (*e.g.*, substitution signatures 3 and 8, and rearrangement signatures 3 and 5) [2,18], loss of heterozygosity (LOH) [19], telomeric-allelic imbalance (TAI) [20], and large-scale state transition (LST) [21]. Two FDA-approved assays integrate tumor BRCA status testing with either an estimate of the percentage of genomic sub-chromosomal LOH (FoundationOne CDx, Foundation Medicine) or a genomic instability score (GIS) which is the overall score summarizing TAI, LST, and LOH (myChoice CDx, Myriad Genetics). Notably, GIS represents the sole genomic scar biomarker studied in first-line randomized controlled trials [9,22].

Current next-generation sequencing (NGS)-based HRD assays employ single nucleotide polymorphisms (SNPs) distributed across the human genome for this purpose. However, the varying methodologies for SNP selection and allele-specific copy number variation (ASCNV) detection pose substantial challenges for accurate patient stratification [23,24]. The HRD Harmonization Project, led by the Friends of Cancer Research (FoCR), has unveiled pronounced variability in inter-assay agreement on homologous recombination (HR) status. Leveraging datasets from 348 ovarian cancer cases, 11 assay developers reported HRD-positive rates spanning from 9% to 67% [25]. Further investigation into 90 ovarian cancer samples by 13 assays revealed HRD-positive rates fluctuating between 23% and 74% [26]. Interestingly, the variability in the in silico analysis was less influenced by the factors included in algorithms, whereas in the latter, the inclusion of certain “consequences” appeared to be a driving factor. A higher consistency was also noted in the measurement of “causes” compared to “consequences”. These findings emphasize the imperative for rigorous evaluation of GIS, a critical and complex biomarker in determining HRD status.

Central to the accurate estimation of GIS is the precise detection of ASCNV, a process that is in dire need of standardization [27–30]. Reference materials and relevant quality control (QC) metrics are required for quality assessment of such SNP-based and ASCNV-based HRD tests. The MicroArray/Sequencing Quality Control (MAQC/SEQC) consortia have established genome-wide reference materials for oncopanel performance assessment, including a pair of DNA derived from matched tumor and normal cell lines of a breast cancer patient, plus a mix of DNA from ten cancer cell lines [31,32]. The corresponding reference datasets cover extensive tumor genomic features like somatic single nucleotide variants (SNVs), small insertions and deletions (indels), copy number variations (CNVs), structural variants, and tumor mutational burden [31–37]. However, these benchmarks do not adequately address the validation needs for HRD detection, particularly due to their limited focus on ASCNV and HRD-related regions. Moreover, given that HRD status is determined based on GIS and the thresholds are subject to validation, the inclusion of reference samples with a broad range of GIS magnitudes is crucial to thoroughly evaluate the analytical performance of HRD assays [38].

In phases IIa and IIb of the Chinese HRD Harmonization Project, we sought to standardize NGS-based HRD detection by constructing a set of DNA reference materials derived from ten paired tumor–normal cell lines accompanied by ASCNV reference datasets and HRD reference scores. These reference materials were orthogonally validated through the whole-genome sequencing (WGS) platform and used to evaluate the performance of four HRD panel assays. We anticipate that this initiative will provide a readily accessible resource for ASCNV standardization and offer reliable tools to enhance the consistency and performance of NGS-based HRD testing.

## Results

### Study design

**Figure 1:**
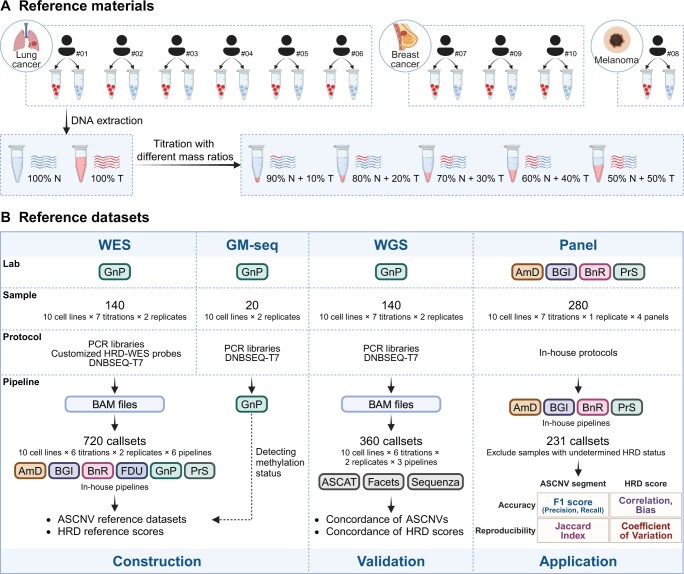
Study design **A**. Establishment of HRD reference materials. Genomic DNA, isolated from ten matched pairs of human cancer and B-lymphocyte cell lines, was mixed at seven mass ratios to create reference materials. **B**. Construction, validation, and application of HRD reference datasets. Employing the reference materials, we generated WES datasets of 140 samples, GM-seq datasets of 20 samples, WGS datasets of 140 samples, and panel datasets of 280 samples. WES, GM-seq, and WGS data were processed by standardized protocols and then analyzed using different pipelines. In contrast, panel sequencing and subsequent bioinformatics analyses were conducted across four independent vendors. We constructed high-confidence ASCNV datasets from WES data and assigned HRD reference scores accordingly. WGS data served to orthogonally validate the reference datasets, which subsequently underpinned the evaluation of four HRD assay panels for accuracy and reproducibility. HRD, homologous recombination deficiency; WES, whole-exome sequencing; GM-seq, genomic methylation sequencing; WGS, whole-genome sequencing; ASCNV, allele-specific copy number variation; N, normal; T, tumor; AmD, Amoy Diagnostics; BGI, BGI Genomics; BnR, Burning Rock Biotechnology; FDU, Fudan University; GnP, GenePlus; PrS, Precision Scientific.

This study developed DNA reference materials for HRD testing, utilizing ten pairs of immortalized tumor–normal cell lines. The cell lines were derived from six lung cancers, three breast cancers, and one melanoma. Each pair originated from matched tumor tissue and leukocytes of the same patient. Genomic DNA (gDNA) was extracted from these pairs and quantified. Subsequently, tumor gDNA was serially diluted with matched normal gDNA at seven tumor:normal mass ratios (0%, 10%, 20%, 30%, 40%, 50%, and 100%) for each pair. This process yielded 70 reference samples with graded tumor DNA levels enabling systematic characterization (**Figure 1**A).

Comprehensive genomic profiling was performed using customized whole-exome sequencing (WES) targeting core-exonic and panel-specific SNPs, genomic methylation sequencing (GM-seq), whole-genome sequencing (WGS), and panels (Figure 1B). While WES and WGS were instrumental in constructing and validating these reference datasets, GM-seq served to validate the methylation and mutation statuses of 43 HRR genes [39]. Centralization of wet-lab experiments and data preprocessing for WES, GM-seq, and WGS was managed by GenePlus (GnP) (Table S1). The processed WES data (BAM files) were then distributed to six participants [Amoy Diagnostics (AmD), BGI Genomics (BGI), Burning Rock Biotechnology (BnR), Fudan Univeristy (FDU), GnP, and Precision Scientific (PrS)] for ASCNV detection, each employing their proprietary algorithms and SNP loci. Notably, FDU utilized the complete set of HRD-WES probes, whereas other vendors conducted analyses with customized SNP subsets via their optimized pipelines. Sequenza, ASCAT, and Facets were used by PrS to analyze the WGS data.

Ultimately, a total of 720 WES callsets and 360 WGS callsets were generated across the ten cell lines, incorporating six tumor titrations with two technical replicates each and involving six pipelines for WES and three for WGS. Based on reliable callsets filtered by mass ratio, purity, ploidy, and CNV coverage rate on autosomes, we constructed high-confidence ASCNV reference datasets following the principle of consensus voting and obtained HRD reference scores.

Using these established reference materials and reference datasets, we assessed the performance of HRD panels from four vendors (AmD, BnR, BGI, and PrS) (Figure 1B). A total of 280 samples were sent to these vendors, with 231 callsets returned for analysis. We excluded normal-derived callsets and nine datasets that could not determine HRD status. The focus of the assessment was on the accuracy of ASCNVs and HRD scores, as well as the reproducibility of these metrics across gradient samples within the same cell line.

### ASCNV irreproducibility in HRD regions

To advance HRD standardization, we undertook a comprehensive analysis of the consistency in ASCNV detection. The Jaccard Index was employed as the metric for this assessment. We focused on three HRD-related variant categories, *i.e.*, LOH, LST, and TAI, all interpreted by scarHRD. Moreover, we examined concordance across various resolution levels, including allele-specific copy number (ASCN), total copy number (CN), allele-specific copy number status (ASCNS), total copy number status (CNS), and detected regions.

**Figure 2:**
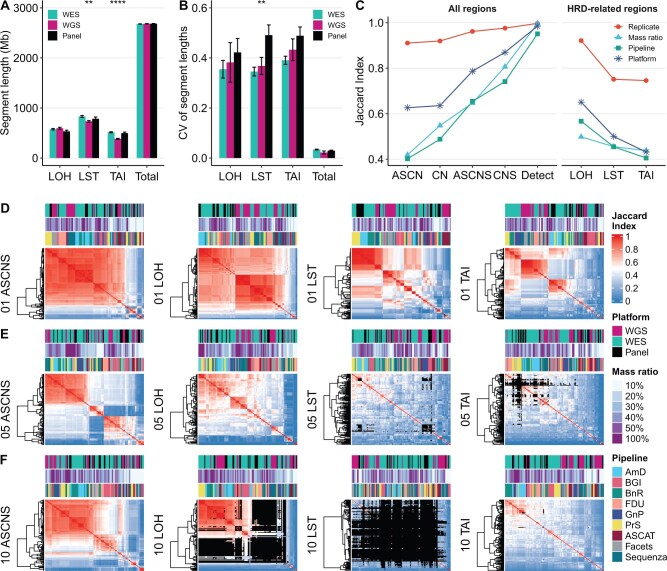
Concordance of CNV detection across platforms, pipelines, mass ratios, and genomic regions **A**. Bar plot showing the length distribution of ASCNV segments for LOH, LST, TAI, and the aggregate regions ascertained by WES, WGS, and panel methodologies. The number of data instances (*n*) used to derive statistics was as follows: WES, *n* = 720; WGS, *n* = 360; panel, *n* = 240. Data are presented as mean ± SD. The *P* values were determined by ANOVA tests with FDR correction (****, *P* < 0.0001; **, *P* < 0.01). **B**. Bar plot showing the CV values of segment lengths with the same statistical approach and annotations. **C**. The median Jaccard Index of ASCN, CN, ASCNS, CNS, detected region (Detect) of the whole-genome regions, as well as the median Jaccard Index of LOH, LST, and TAI of the HRD-related regions. The comparisons spanned technical replicates (*n* = 1080), mass ratios (*n* = 6510), analytical pipelines (*n* = 4986), and platforms (*n* = 462), with only the compared conditions being variables. *n* signifies the count of pairwise comparisons. **D**.–**F**. Heatmaps showcase the concordance of ASCNS, LOH, LST, and TAI across cell lines 01 (D), 05 (E), and 10 (F), utilizing three different platforms and nine pipelines across six mass ratios. The black area marks instances where the target variant was undetected in one or both samples within the pairwise comparisons. LOH, loss of heterozygosity; LST, large-scale state transition; TAI, telomeric-allelic imbalance; ASCN, allele-specific copy number; CN, total copy number; ASCNS, allele-specific copy number status; CNS, total copy number status; FDR, false discovery rate; CV, coefficient of variation; SD, standard deviation.

Our findings revealed generally uniform ASCNV detection across different platforms over the entire detectable regions, with notable exceptions in HRD-related regions. In these HRD-related areas, WES and panels were more proficient in identifying longer fragments of LST and TAI compared to WGS. This suggests that incorporating SNP backbones may effectively capture specific CNV types (**Figure 2**A). WES exhibited least variability, followed by WGS, while panels displayed the highest variability, potentially due to the variabilities inherent in the experimental procedures of panel assays (Figure 2B). Compared to WGS, WES also exhibited higher consistency in LOH, LST, and TAI regions, also demonstrating the superiority of utilizing WES probes (Figure S1).

In terms of reproducibility, especially in HRD-related regions, challenges were evident. Our assessment spanned from simple interval comparisons to complex ASCN evaluations, which required stringent concordance between both alleles. This inevitably led to diminished consistency, as depicted in Figure 2C. Despite LOH and TAI analyses relying primarily on relative differences between major and minor alleles, making them somewhat less reliant on absolute copy number accuracy, they still demonstrated varying levels of reproducibility. LOH regions showed the highest consistency, followed by LST regions (where chromosomal breakpoint detection introduced additional challenges) and TAI regions (which were further complicated by complex chromosomal rearrangements) (Figure S2).

The complexity of HRD regions, often involving large-scale structural variations such as deletions, duplications, and rearrangements, introduces challenges for ASCNV detection by increasing background noise and the likelihood of false positives or negatives. The limitations of short-read NGS technologies further complicate both the accurate determination of ASCNs and the detection of chromosomal breakpoints. These factors lead to reduced consistency in ASCN detection and make it difficult to reach consensus across pipelines. The need for concordance between ASCNs further reduces accuracy and reproducibility.

To address potential biases, we implemented a consensus voting approach across multiple pipelines and technical replicates, focusing on ASCNS at the whole-genome level where higher consistency was observed. Regions with significant ambiguity or complex rearrangements were excluded to avoid unreliable results. Although this conservative approach led to the exclusion of some complex regions, it enhanced the reliability of the reference datasets for future HRD testing applications.

### Cell line-specific challenges and ploidy implications

The genomic individuality of different cell lines was evident. In cell line 01, the consistency in detecting ASCNS and LOH was comparable, but it was noticeably reduced for LST and TAI, with results clustering according to the analytical process (Figure 2D). In contrast, cell line 05 exhibited considerable inconsistency in ASCNS across the whole genome, with overall consistency for LST and TAI generally below 0.5, including instances where some processes failed to detect any variations (Figure 2E). More complex still, cell line 10, while showing better consistency in ASCNS compared to cell line 05, had numerous undetected instances in LOH and even more in LST (Figure 2F).

Platform and pipeline preferences also varied across different cell lines (Figure S3). Regardless of the genomic interval assessed or the level of precision, we found that an adequate tumor:normal mass ratio is fundamental for ensuring reliable ASCNS results. Low-purity samples exhibited almost no reproducibility. Certain cell lines exhibited extremely low consistency across different mass ratios, such as cell lines 02, 04, 07, and 08 (Figures S2 and S3).

Interestingly, we observed that differences in the interpretation of ploidy in hyperploid genomes by different analytical processes affected the consistency of ASCNV detection and the accuracy of purity estimation. Particularly at the genome level, there was a significant negative correlation between ploidy differences and ASCNS consistency across different processes, which was not significant for LOH, LST, and TAI. This is likely because the interpretation of these three types of variations is not influenced by deviations in copy numbers caused by ploidy (Figure S4). Moreover, for hyperploid genome samples, we also observed deviations between the purity calculated from sequencing data and the theoretical values of mass ratios, with higher ploidy correlating with lower estimated purity (Figure S5).

### Construction of HRD reference datasets

**Figure 3:**
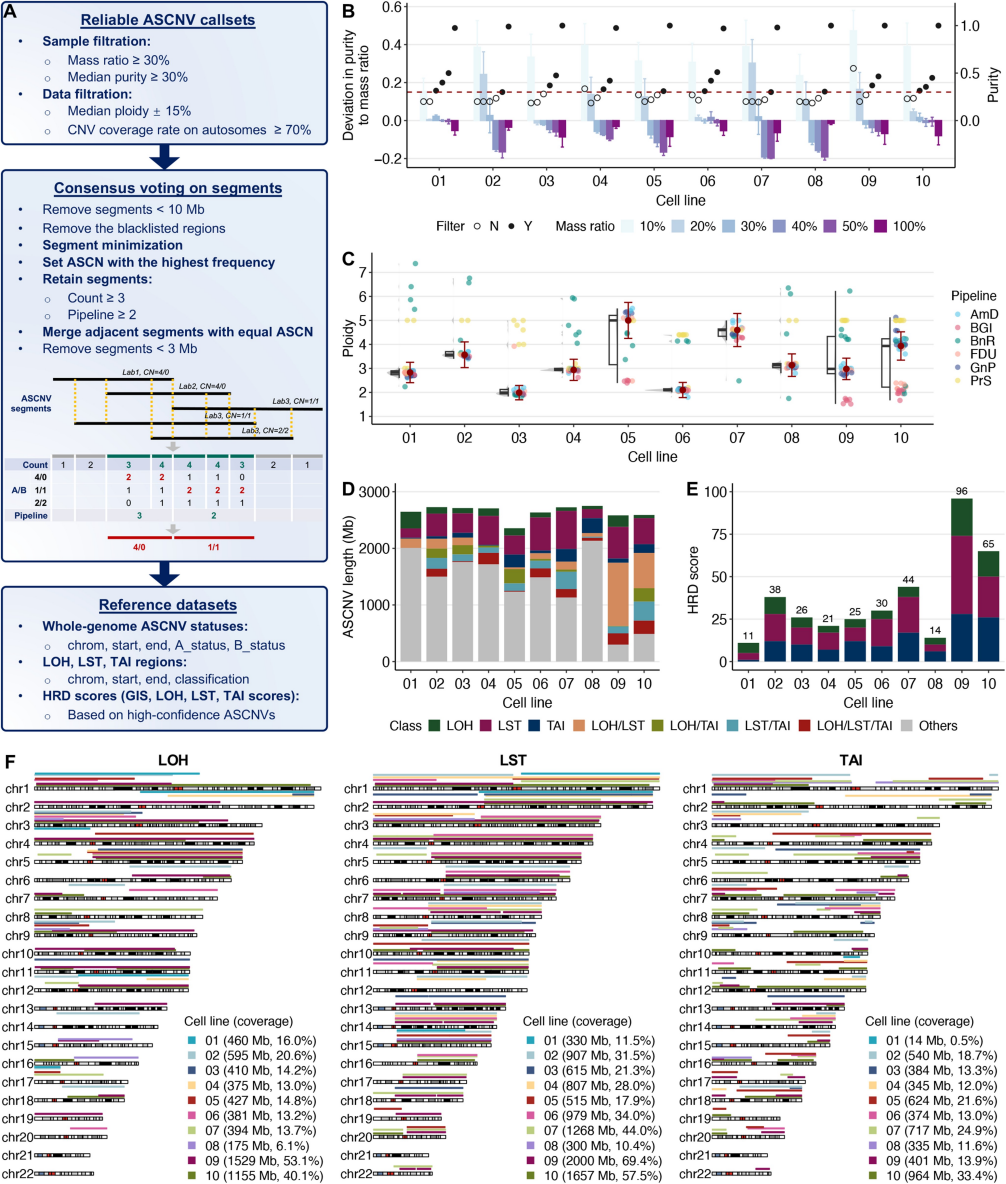
Definition of HRD reference datasets **A**. Reference dataset construction workflow. The process entailed identifying reliable ASCNV calls, achieving consensus on segment designation, and delineating high-confidence genome-wide ASCNVs. This was followed by cataloging HRD-related regions (*i.e.*, LOH, LST, and TAI) and assigning HRD-related scores (*i.e.*, GIS, LOH, LST, and TAI). **B**. Bar plot showing the deviation in purity from mass ratio (left y-axis) and point plot showing the median purity (right y-axis) detected from six pipelines. The deviations are presented as mean ± SD. Solid circles highlight samples retained (Y) based on mass ratio, while open circles delineate excluded samples (N) below 30% purity (indicated by the red dotted line). **C**. Box and violin plots on the left indicate the spread of ploidy data, with medians shown by lines within the boxes and whiskers extending to 1.5× the interquartile range. The box plots on the right highlight median ploidy values (marked by solid red circles), with whiskers capturing the upper and lower 15% of the range utilized for developing the reference datasets. **D**. Stacked bar plot quantifies the lengths of LOH, LST, and TAI regions and their intersections, as well as the lengths of regions not directly implicated in HRD scoring of the ASCNV reference datasets. **E**. Stacked bar plot showing the reference GIS (shown above each bar) and the constituent LOH, LST, and TAI scores. **F**. Chromosome view of high-confidence LOH, LST, and TAI regions across the ten cell lines. GIS, genomic instability score.

In the development of reference datasets, we employed a structured three-step methodology, as depicted in **Figure 3**A. The initial phase involved the selection of ASCNV callsets, guided by criteria including mass ratio, purity, ploidy, and CNV coverage rate on autosomes. We established a threshold of retaining only samples with purity ≥ 30%. However, this threshold varied among different cell lines due to differences observed between purity and DNA mass ratio. This discrepancy may be caused by the quality of CNV calls and the accuracy of ploidy estimation across cell lines [37]. Specifically, for cell lines 01, 06, and 10, a minimum purity of 30% corresponded to a DNA mass ratio of 30% or more. In contrast, for cell lines 03, 04, and 09, this purity corresponded to a DNA mass ratio of at least 40%, and for cell lines 02, 05, 07, and 08, the required ratio was 50% or more (Figure 3B). This is consistent with our prior observations that hyperploid genome samples exhibit greater ploidy variation across pipelines, as well as greater purity bias (Figures S4 and S5). Consequently, we opted to retain data within a 15% range of the median ploidy value (Figure 3C). Following this criterion, we considered all ASCNV callsets with CNV coverage ≥ 70% on autosomes, resulting in 250 reliable ASCNV calls.

The subsequent stage encompassed selection and segmentation, supplemented by a frequency-based voting mechanism. Fragments detected in only a single process, as well as those within identified blacklisted regions, were excluded. Finally, two categories of HRD reference datasets were established. The first category comprised ASCNV reference datasets. At the whole-genome level, this involved defining regions of high-confidence ASCNVs, along with the classification of major and minor allele statuses. Within HRD-specific regions, we identified high-confidence segments for LOH, LST, and TAI, excluding the specification of statuses or copy numbers. The second category comprised HRD reference scores derived from these high-confidence ASCNVs. For each cell line, we computed reference GIS and their individual LOH, LST, and TAI components (**Table 1**).

**Table 1:** Characteristics of the reference materials and reference datasets

Cell line	Cancer type	Ploidy	ASCNV (%)	LOH region (%)	LST region (%)	TAI region (%)	LOH score	TAI score	LST score	GIS
01	Non-small cell lung carcinoma	2.8	2646 Mb (91.84)	460 Mb (16)	330 Mb (11.5)	14 Mb (0.5)	6	1	4	11
02	Large cell lung carcinoma	3.6	2719 Mb (94.37)	595 Mb (20.6)	907 Mb (31.5)	540 Mb (18.7)	10	12	16	38
03	Small cell lung carcinoma	2.0	2701 Mb (93.76)	410 Mb (14.2)	615 Mb (21.3)	384 Mb (13.3)	6	10	10	26
04	Small cell lung carcinoma	2.9	2706 Mb (93.93)	375 Mb (13.0)	807 Mb (28.0)	345 Mb (12.0)	4	7	10	21
05	Lung adenocarcinoma	5.1	2340 Mb (81.22)	427 Mb (14.8)	515 Mb (17.9)	624 Mb (21.6)	5	12	8	25
06	Small cell lung carcinoma	2.1	2630 Mb (91.31)	381 Mb (13.2)	979 Mb (34)	374 Mb (13.0)	5	9	16	30
07	Breast ductal carcinoma	4.6	2647 Mb (91.90)	394 Mb (13.7)	1268 Mb (44.0)	717 Mb (24.9)	6	17	21	44
08	Cutaneous melanoma	3.1	2749 Mb (95.43)	175 Mb (6.1)	300 Mb (10.4)	335 Mb (11.6)	4	6	4	14
09	Breast ductal carcinoma	2.9	2549 Mb (88.49)	1529 Mb (53.1)	2000 Mb (69.4)	401 Mb (13.9)	22	28	46	96
10	Breast ductal carcinoma	4.0	2549 Mb (88.47)	1155 Mb (40.1)	1657 Mb (57.5)	964 Mb (33.4)	15	26	24	65

*Note*: % indicates the percentage of length of ASCNV segments/LOH regions/LST regions/TAI regions *vs.* the total autosomal length of the reference genome 37d5. ASCNV, allele-specific copy number variation; LOH, loss of heterozygosity; LST, large-scale state transition; TAI, telomeric-allelic imbalance; GIS, genomic instability score; Mb, million bases.

The lengths of high-confidence ASCNVs across the ten cell lines varied significantly, with cell lines 07, 09 and 10, (all of which are breast cancer lines) displaying the highest proportion of HRD regions (60.7%, 89.6%, and 83.1%, respectively). These cell lines also harbored mutations associated with defective BRCA1/2 function, with cell line 10 additionally showing *BRCA2* methylation silencing (Tables S2−S3). Some CNVs simultaneously fulfilled the definitions of LOH, LST, and TAI, resulting in eight possible combinations [16]. This phenomenon of CNV category overlap was also evident in our constructed reference dataset (Figure 3D). The GIS values for these reference materials varied from 11 to 96, encompassing the typical range observed in clinical HRD testing (Figure 3E). By a singular analysis of specific chromosomal regions for LOH, LST, and TAI, the LOH regions covered 20 autosomes, LST involved 22 autosomes, and TAI involved 21 autosomes, with each cell line presenting distinct variations. This approach enabled a more thorough evaluation of ASCNV detection accuracy (Figure 3F).

### Orthogonal validation of HRD reference datasets via WGS platform

An orthogonal validation of HRD reference datasets was carried out using the WGS platform. We assessed the congruence between WES-derived reference datasets and WGS results, focusing on two aspects: ASCNV status and HRD scores. Three open-source tools, *i.e.*, ASCAT, Facets, and Sequenza, were employed for ASCNV detection. ASCAT primarily focuses on purity and ploidy estimation by analyzing B-allele frequency (BAF) and logR signals across the genome, making it sensitive to purity variations and ideal for samples with lower or mixed purity levels [40]. Sequenza, on the other hand, uses a maximum likelihood approach, which allows it to handle complex genomic structures and variable ploidy levels effectively, making it particularly useful in highly heterogeneous samples [41]. Facets employs a Bayesian framework with iterative optimization, excelling in low-purity or noisy samples where consistency is critical [42]. Thus, ASCAT is effective in purity-sensitive contexts, Sequenza excels at managing complex ploidy, and Facets is reliable in low-purity environments. By combining these tools, the overall detection accuracy is enhanced, facilitating an objective validation of the reference datasets.

In highly unstable cell lines, such as 07, 09, and 10, significant variability in HRD scores was observed across mass ratios. ASCAT exhibited larger differences in GIS between high and low purity across all cell lines, likely due to its sensitivity to purity variations (Figure S6). Similarly, in evaluating consistency with the ASCNV reference dataset, a reduced proportion of common fragments detected by different callers was noted at lower purities (Figure S7). Therefore, sample selection criteria were used in the subsequent validation and HRD panel performance assessment. Cell lines 02, 05, and 07 had high ploidy and high purity bias, requiring DNA mass ratios of 40% or greater, whereas the threshold for the other cell lines was 30%.

**Figure 4:**
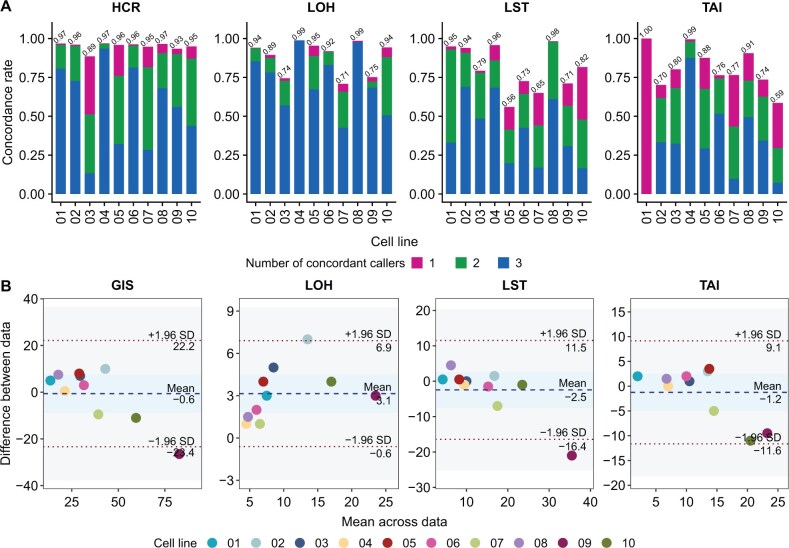
Orthogonal validation of HRD reference datasets via WGS platform **A**. Stacked bar plots showing the proportion of concordant regions between the reference datasets and WGS-derived ASCNV segments. The colors represent the number of callers (ASCAT, Facets, and Sequenza) that concordantly identified the regions: pink (one caller), green (two callers), and blue (three callers). Samples with mass ratios below 40% for cell lines 02, 05, and 07 and below 30% for other cell lines were excluded. **B**. Bland-Altman plots for agreement analysis between HRD reference scores and WGS-derived scores. LoAs are shown as red dotted lines with 95% CI (gray areas). Biases are shown as blue dotted lines with 95% CI (blue areas). Statistics for WGS-based HRD-related scores were based on median values of the samples that met the mass ratio criteria. HCR, high-confidence region; LoA, Limit of Agreement; CI, confidence interval.

The ASCNV reference datasets for all ten cell lines were validated to some extent (**Figure 4**A). Notably, in the overall high-confidence regions (HCRs), without distinguishing between analytical pipelines, 89%–97% were detectable in WGS. LOH reference datasets reached 89%–99% concordance in most cell lines, except for cell lines 03, 07, and 09, which showed 71%–75% concordance. However, LST and TAI reference datasets exhibited over 85% concordance only in four cell lines, with lower consistency in others. This underscores the need for a comprehensive evaluation of HRD-related ASCNVs using the complete set of the ten reference datasets.

Regarding HRD-related scores, the references and WGS-derived scores demonstrated strong consistency (Figure 4B). The Bland-Altman analysis revealed a mean bias of −0.6 between reference and WGS-derived GIS values with Limits of Agreement (LoAs) of −23.4 to 22.2, a mean bias of 3.1 with LoAs of −0.6 to 6.9 for LOH, a mean bias of −2.5 with LoAs of −16.4 to 11.5 for LST, and a mean bias of −1.2 with LoAs of −11.6 to 9.1 for TAI. Although GIS and LST scores for cell line 09 fell outside the lower LoA, they remained within the 90% confidence interval (CI) of LoAs. Most importantly, GIS and its sub-scores showed no significant discrepancies between WGS measurements and reference datasets, confirmed by one-sample *t*-tests (*P* > 0.05), indicating zero within the 95% CI as shown in the blue regions.

### Performance of HRD panel assays

To assess the performance of HRD panel assays, we utilized the HRD reference materials and datasets for comprehensive evaluation. The reproducibility of ASCNVs across varying mass ratios within the same cell line was measured using the Jaccard Index, while ASCNV accuracy was assessed using the F1 score. The coefficient of variation (CV) was employed to gauge the reproducibility of HRD scores, and the accuracy of HRD scores was evaluated using Spearman correlation coefficients, deviation analysis, and Bland-Altman analysis.

**Figure 5:**
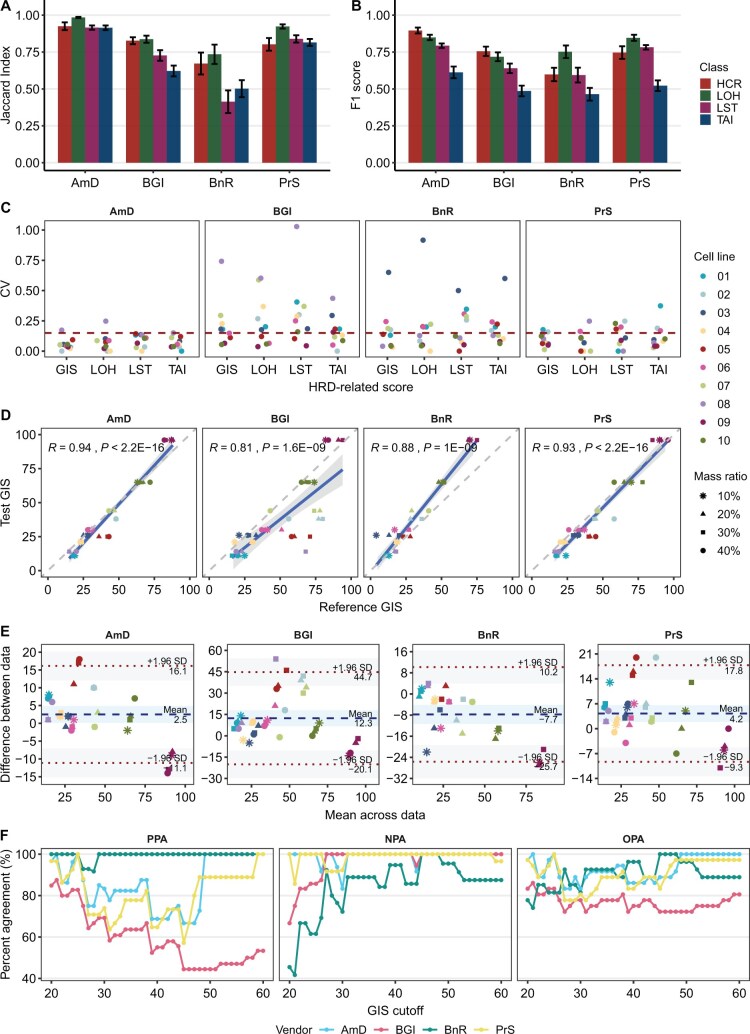
Accuracy and reproducibility of HRD panel assays **A**. Bar plot showing the Jaccard Indices for ASCNV status, LOH, LST, and TAI regions across different mass-ratio samples for each cell line. **B**. Bar plot showing F1 scores for ASCNV status, LOH, LST, and TAI regions based on reference datasets. Data are presented as mean ± SD. **C**. CV values of HRD-related scores (GIS, LOH, LST, and TAI) of different mass-ratio samples for each cell line, with a 15% threshold indicated by a red dashed line. **D**. Scatter plots of the reference and panel-derived GIS values. *R* denotes the Spearman correlation coefficient. Each point represents one sample; solid lines indicate fitted lines obtained from linear regression, with shading indicating 95% CI. **E**. Bland-Altman plots for agreement analysis between reference and panel-derived GIS values. LoAs are shown as red dotted lines with 95% CI (gray areas). Biases are shown as blue dotted lines with 95% CI (blue areas). **F**. Effect of GIS thresholds on PPA, NPA, and OPA. In all the abovementioned assessments, samples with mass ratios below 40% were removed for cell lines 02, 05, and 07, and samples with mass ratios below 30% were removed for other cell lines. The number of samples (*n*) used to derive statistics was as follows: AmD, *n* = 36; BGI, *n* = 36; BnR, *n* = 27; PrS, *n* = 36. Nine samples tested by BnR returned “undetermined” results and were therefore not included. PPA, positive percent agreement; NPA, negative percent agreement; OPA, overall percent agreement.

Regarding ASCNV reproducibility, AmD excelled in identifying HCR copy number status and in classifying LOH, LST, and TAI regions, achieving a Jaccard Index range of 0.91–0.98. This performance was followed by PrS (0.80–0.92), BGI (0.62–0.84), and BnR (0.41–0.50) (**Figure 5**A). The lower reproducibility observed in BnR was primarily due to its underperformance in cell lines 02, 05, and 07, especially in HCR, LOH, and LST, coupled with generally poor reproducibility in TAI. BGI, while showing lower reproducibility than AmD and PrS in these cell lines, did not display a clear cell line preference (Figure S8).

Regarding ASCNV accuracy, AmD outperformed the others (F1 score = 0.61–0.90), with the remaining vendors ranked similarly but with minor differences: PrS (F1 score = 0.52–0.85), BGI (F1 score = 0.49–0.76), and BnR (F1 score = 0.46–0.75) (Figure 5B). BGI and BnR’s lower accuracy in certain cell lines paralleled their reproducibility (Figure S9). The total fragment lengths detected by the panels were largely consistent with the HCR total length, exhibiting similar precision and recall. However, disparities became apparent in the LOH, LST, and TAI regions (Figure S10). The BGI panel showed a significantly lower precision than recall in LST, and PrS displayed a similar pattern in TAI. This suggested that both panels detected a large number of fragments that did not match the reference dataset, which could be potential false positives (Figure S11). BnR displayed higher precision relative to recall, suggesting that many segments in the reference datasets might have been missed (Figures S10 and S11).

For HRD score reproducibility, the percentage of cell lines with GIS values within a 15% CV was 90% for AmD, 80% for PrS, 60% for BnR, and 40% for BGI (Figure 5C). The Spearman correlation ranking for HRD scores mirrored the reproducibility ranking: AmD (*R* = 0.94), PrS (*R* = 0.93), BnR (*R* = 0.88), and BGI (*R* = 0.81) (Figure 5D). The GIS distributions of AmD and PrS closely mirrored the reference scores. BGI’s GIS values were lower, largely attributed to less frequent detection of LOH and LST, while BnR’s GIS values were elevated, particularly at higher GIS levels, due to biases in LST and TAI detection (Figure S12). Overall, there was a statistically significant correlation between high HRD score bias with low ASCNV accuracy (Figure S13). In addition, Bland-Altman analysis corroborated these rankings, with mean biases of AmD at 2.5, PrS at 4.2, BnR at −7.7, and BGI at 12.3, and a similar number of samples falling outside the LoAs for each: AmD (4), PrS (3), BnR (2), and BGI (2). Contrasting with WGS, significant measurement differences between the four panels and the WES-derived reference GIS values were confirmed by one-sample t-tests (*P* < 0.05), indicating zero outside the 95% CI (as shown in the blue regions in Figure 5E).

Considering that HRD testing fundamentally involves qualitative assessment based on GIS, we further explored the performance of four panels at different thresholds (Figure 5F). Notably, despite AmD showing higher consistency with the reference datasets in both ASCNV and GIS, no marked differences were observed in qualitative assessments compared to the overall percent agreement (OPA) of BnR and PrS. For instance, BnR consistently exhibited high positive percent agreement (PPA), but its negative percent agreement (NPA) was significantly lower than the other panels at most thresholds. In contrast, AmD, BGI, and PrS consistently showed high NPA.

## Discussion

As part of the Chinese HRD Harmonization Project, we have enabled a nuanced and precise characterization of HRD-related ASCNVs across diverse genomic landscapes. In pursuit of establishing a benchmark for NGS-based HRD detection, we created reference samples from ten human tumor cell lines and their corresponding normal cell lines, and established reference datasets for ASCNV and HRD scores (Figure 1). The reference materials, covering a broad HRD score range, allow for the validation of assays at various detection thresholds. The ASCNV reference datasets cover a large number of HRD-related regions and show overall consistency in the orthogonal validation process (Figures 3 and 4). This provides a reliable tool for standardizing HRD scores based on genomic scar detection.

Our research reveals the intricate challenges and imperative need for standardization in HRD testing. The unique patterns of different cell line genomes and the significant impact of tumor ploidy on ASCNV detection introduce additional complexity to the analysis (Figure 2). We also draw attention to the disparities in ploidy interpretation across different analytical pipelines, which significantly affect the consistency of ASCNV and purity estimation (Figures S4 and S5). Therefore, it is important to consider genomic composition and ploidy variation when developing, evaluating, and using analysis software packages.

Furthermore, the observed performance discrepancies in HRD panel assays, particularly in HRD-specific regions, signal an urgent need for more rigorous standardization of detecting CNVs in clinical and research settings (Figure 5). Accurate prediction of GIS is closely related to the ability to detect CNV, which is measured in this study using ASCNV status. The more accurately CNV is detected, the more reliable GIS tends to be (Figure S13). In particular, we found that the HRD scores generated by the Myriad MyChoice assay used by BnR were consistently higher than the reference GIS (Figure 5D). This discrepancy was primarily attributed to the elevated scores in two HRD-positive samples, 09 and 10 (Figure S12). The trends of PPA and NPA in HRD classification of BnR were also different from the other three panel assays. These findings suggest a systematic bias inherent in the reference datasets with Myriad reagents (Figure 5F).

A key challenge identified in our study is the establishment of stratified thresholds of GIS for distinguishing between positive and negative cases, which is consistent with the findings of the Friends’ HRD Harmonization Project [25,26]. We also note a distinction between quantitative assessment based on reference GIS and qualitative assessment of HRD assays (Figure 5F). The thresholds used to determine HR status in individual assays may vary across cancer types, emphasizing the need for a more refined assessment framework when performing qualitative assessments [15,43]. Future research should prioritize qualitative evaluations to refine the precision and applicability of HRD assessments in cancer treatment.

In our initiative, we have prioritized the evaluation of LOH, LST, and TAI indices to minimize assay variability and expedite the development of clinically applicable products. In contrast, the Friends’ HRD Harmonization Project incorporated up to 17 assays that employed at least eight distinct computational methods [25,26]. Their latest findings emphasize the significance of establishing standardized reference materials, which is crucial for setting a gold standard in HRD testing. Tackling the essential aspects of assay consistency, both projects support the notion that creating reference materials is not only feasible but also essential for refining HRD testing.

While our research primarily assesses the analytical performance of ASCNV detection, clinical efficacy remains unaddressed. The selected cell lines cover a wide range of genomic instability, but their representativeness is limited. Although small cell lung cancer patients with HRD may respond to PARPis and immuno-neoadjuvant therapy, those with confirmed efficacy significance remain primarily ovarian, breast, pancreatic, and prostate cancers [44,45]. Given that the primary aim of this study is to evaluate analytical performance, the chosen cancer cell lines provide an appropriate genomic context for confirming assay accuracy and reproducibility. The ongoing phase III of the Chinese HRD Harmonization Project focuses on advanced ovarian cancer clinical samples, with comprehensive efficacy data correlating HRD detection to drug response. Further work is needed to evaluate the consistency between cell line-based reference materials and clinical samples.

Looking forward, the standardization of HRD detection at the genomic level is urgently needed. Integrating various HRD components using algorithms such as scarHRD, HRDetect, and CHORD may evolve as the gold standard in identifying genomic scars, offering a more comprehensive assessment than mutation-based methods alone [46–48]. Moreover, further investigation is required to determine whether and how genome methylation levels affect HRD status and CNV distribution, as these could significantly refine the accuracy of HRD detection. Incorporating the effects of gene mutations and methylation, along with other types of genomic scars, will be essential to optimizing reference datasets and establishing robust benchmarks for HRD testing.

In summary, the Chinese HRD Harmonization Project has established an array of meticulously characterized DNA reference materials and datasets, setting a crucial benchmark for evaluating HRD assays. The thorough examination of four HRD panels against these standards has unveiled disparate performances among various assays, highlighting an ongoing need for enhancement and standardization of HRD testing methodologies. As the field of HRD testing advances, these reference materials and datasets will be pivotal in ensuring the precision, reliability, and consistency of HRD assays.

## Materials and methods

### Preparation of reference materials

#### Selection and identification of cell lines

To prepare the candidate reference samples, we selected ten pairs of tumor–normal matched immortalized cell lines from GeneWell (Shenzhen, China). The selection encompassed a diverse range of cancers, including three small cell lung carcinomas, one non-small cell lung carcinoma, one large cell lung carcinoma, one lung adenocarcinoma, three breast ductal carcinomas, and one cutaneous melanoma (GW-FGTM003, GW-FGTM004, GW-FGTM005, GW-FGTM006, GW-FGTM002, GW-FGTM008, GW-FGTM009, GW-FGTM010, GW-FGTM011, and GW-FGTM012). Each pair originated from a single patient, comprising a tumor-derived line and its leukocyte-derived normal counterpart. The selection criteria prioritized GIS representation of each cancer type and diversity in GIS, LOH, TAI, and LST across cell lines.

#### DNA extraction and quality assessment

The gDNA was extracted using the TANBead Nucleic Acid Extractor (Catalog No. 301132, TANBead, Taoyuan, China). DNA purity was assessed by measuring the A260/A280 ratio using NanoDrop 8000 spectrophotometry (Thermo Fisher Scientific, Wilmington, DE), and DNA concentration was quantified using Qubit 4.0 fluorometer (Invitrogen, Carlsbad, CA. DNA quality thresholds were set at a NanoDrop-to-Qubit (N/Q) ratio of < 6 and a concentration of > 100 ng/µl for downstream experiments.

#### Configuration of tumor titration series

The gDNA from the ten cell-line pairs was diluted to a uniform concentration of 50 ng/µl. Tumor and normal DNA samples from each pair were mixed in specific mass ratios, creating a series of tumor DNA proportions (100%, 50%, 40%, 30%, 20%, 10%, and 0%) and corresponding leukocyte DNA proportions (0%, 50%, 60%, 70%, 80%, 90%, and 100%).

### Design of HRD-WES probes

To accurately assess HRD, we addressed the variable SNP distribution across vendors by combining SNP regions from six providers: AmD, BnR, BGI, PrS, GnP, and Teddy Clinical Research Laboratories, creating a set of 23-Mb candidate regions. From these, 12-Mb target regions were selected for online probe design through RocheHyperDesign, resulting in probes covering 16 Mb within the SNP backbone. To ensure comprehensive exon region coverage, these SNP backbone probes were integrated with IDT xGen Exome Research Panel v2.0 probes, forming a 58-Mb probe collection designated as HRD-WES probes. This probe set covered about 19.1 Mb, with uniform distribution and a small percentage of intervals exceeding 100 kb, making it suitable for HRD detection. The capture efficiency of these probes was approximately 60%, with other quality metrics including average coverage and GC content falling within acceptable ranges.

### WES and analysis

A total of 800 ng of each DNA sample was fragmented using a LE220 sonication system (Covaris, Woburn, MA) and then subjected to end repair, A-tailing, and adapter ligation with the Hieff NGS Ultima DNA Library Prep Kit for MGI (Catalog No.13310ES98, Yeasen Biotech., Shanghai, China) according to the manufacturer’s protocol. Approximately 1000 ng of prepared DNA in a volume of 3.5 µl was then captured using the customized HRD probes, followed by amplification of the captured library with indexing primers. QC was performed using the LabChip GX Touch (PerkinElmer, Waltham, MA) with a DNA chip. After quantification with a Qubit 4.0 fluorometer (Invitrogen), the libraries were sequenced on the DNBSEQ-T7 platform (MGI Tech, Shenzhen, China), and 150 bp paired-end reads were generated as FASTQ files with four libraries per lane.

Bioinformatic analysis began with the removal of low-quality reads and adapters using Fastp (v0.20.0) with the parameters “-f 10 -F 10 -q 5 -u 50 -n 14”. Sequence alignment was performed using BWA (v0.7.17) with the hs37d5 reference genome and alignment parameters of 4.1. Post-alignment processing was conducted using SAMtools (v1.16) to generate sorted and indexed BAM files. Six participants (AmD, BGI, BnR, GnP, FDU, and PrS) performed ASCNV detection using these BAM files. FDU directly utilized all HRD-WES probes without additional modifications to these BAM files, whereas the other five participants performed personalized SNP selection for analysis. The SNP coverage lengths were 1.3 Mb (AmD), 5.5 Mb (BGI), 0.8 Mb (BnR), 5.9 Mb (GnP), 19.1 Mb (FDU), and 2.6 Mb (PrS). In addition, germline variants were detected using DNAscope (Sentieon Genomics, Mountain View, CA), and somatic variants were detected using RealDcaller2, an in-house tool developed by GnP.

### GM-seq and analysis

A total of 300 ng of each DNA sample was fragmented using a LE220 sonication system (Covaris) and then subjected to end repair, A-tailing, and adapter ligation with the Hieff NGS Ultima Pro DNA Library Prep Kit for Illumina (Catalog No. 12201ES96, Yeasen Biotech) following the manufacturer’s protocol. After magnetic bead purification, 5-methylcytosine (5mC) and 5-hydroxymethylcytosine (5hmC) were oxidized to form 5-acylcytosine (5fC) or 5-carboxylcytosine (5caC) using the TET enzyme (Catalog No. E7120L, NEB, Ipswich, MA). Subsequently, 5fC or 5caC was treated with pyridine borane and reduced to dihydrouracil (DHU). To assess the efficiency of the TET enzyme (Catalog No. E7120L, NEB), control samples, including 0.5% synthetic methylated DNA and 1% synthetic unmethylated DNA, were incorporated into the conversion system by mass. The sequencing libraries, barcoded for identification, were synthesized through PCR amplification and sequenced on the DNBSEQ-T7 platform (MGI Tech), generating 150 bp paired-end reads in FASTQ format with five libraries per lane [49].

The pipline from sequencing data to BAM files was consistent with WES. AsTair was employed to detect methylation sites and extract methylation signal-related data [50]. We analyzed potential promoter regions of 43 HRR genes, which were selected based on their covariate relationship with HRD indicators, with a significance threshold of *P* < 0.05 [39]. These promoter regions were defined as 2000 bp upstream of the transcription start sites. The mean thymidine frequency in these regions was calculated, serving as an indicator of gene methylation levels.

### WGS and analysis

The gDNA was prepared by fragmenting 500 ng of each sample using the LE220 sonication system (Covaris). The fragmented DNA underwent end repair, A-tailing, and adapter ligation using the Hieff NGS Ultima Pro DNA Library Prep Kit for Illumina (Catalog No. 12201ES96, Yeasen Biotech) according to the manufacturer’s protocol. Subsequent amplification of each library was conducted using indexing primers. QC was performed using the LabChip GX Touch (PerkinElmer) with a DNA chip. After quantification with a Qubit 4.0 fluorometer (Invitrogen), the libraries were sequenced on the DNBSEQ-T7 platform (MGI Tech), and 150 bp paired-end reads were generated as FASTQ files with two libraries per lane.

WGS data preprocessing mirrored the protocol established for WES, resulting in BAM files. Subsequently, PrS detected ASCNVs using Sequenza [41], a method recommended in a scarHRD-related study [47], based on the SNPs in the whole genome. In addition, ASCAT [40] and Facets [42] were used to mitigate potential analytical bias inherent to single-software approaches.

### HRD panel assays

Aliquots of 70 DNA samples were distributed to AmD, BnR, BGI, and PrS. Each conducted assays independently in their labs using their respective standard operating procedures for HRD assays. BnR employed the Myriad MyChoice CDx assay.

### HRD scores

The definitions of HRD-related terms have been standardized in Phase I of the Chinese HRD Harmonization Project. LOH is defined as the deletion of one of the two allelic genes in a pair of homologous chromosomes. LOH score is calculated based on autosomal chromosome fragments greater than 15 Mb that are exclusively composed of LOH, excluding the total count of such fragments across the entire chromosome. TAI is defined as autosomal segments with an uneven number of alleles in the telomeric region, and TAI score is determined by the number of chromosomal segments which exhibit allelic imbalance starting from the telomere but not exceeding the centromere and are not shorter than 11 Mb. LST is defined as the number of autosomal segments with at least 10-Mb chromosomal breaks between adjacent regions, and a distance between them is no more than 3 Mb [3]. GIS is calculated as the sum of LOH, LST, and TAI scores. The modified version of scarHRD used in this study is available at https://github.com/AmoydxGXP/scarHRD.

### Construction of reference datasets

The reference datasets were constructed using 720 sets of WES data analysis results, including ASCNV callsets, purity, and ploidy. These datasets were derived from six tumor-normal paired analysis pipelines that were applied to ten cell lines, with each cell line analyzed in two technical replicates of six tumor gradients. The reference datasets were constructed through the following steps.

#### Selection of reliable ASCNV callsets

In compliance with prevalent clinical testing standards, we required a minimum tumor purity of 30% in the samples. Datasets pertaining to mass ratios of 10% and 20% were initially excluded. Subsequently, the median of the six purity outcomes at each quality ratio per cell line was adopted as the actual tumor purity, establishing a correlation between quality ratio and tumor purity. Dilution gradients with an actual tumor purity of ≥ 30% were exclusively retained.

Moreover, considering the impact of ploidy on ASCNV, the median ploidy of each cell line was computed, and data within a ±15% range of this median were preserved. Additionally, we evaluated the proportion of detected ASCNV lengths against the total number of autosomal regions, opting to retain callsets with ≥ 70% coverage. Following these criteria, 250 reliable ASCNV callsets were selected for the construction of the reference datasets.

#### Selection of high-confidence ASCNV segments

Firstly, blacklist regions for each cell line were determined, focusing on areas with insufficient SNP counts or CNVs present in control samples. This resulted in the creation of blacklisted intervals, as shown in Table S4, with the cumulative length of excluded regions in each cell line not exceeding 400 Mb. The total lengths of the blacklisted regions excluded in cell lines 01, 04, 05, 06, and 09 were 29 Mb, 76 Mb, 329 Mb, 171 Mb, and 102 Mb, respectively, which accounted for 1%, 3%, 11%, 6%, and 4% of the total autosomal length of the genome.

After excluding regions overlapping with the blacklisted areas and ASCNV segments smaller than 10 Mb, the remaining intervals were organized into an interval tree. This tree was segmented into distinct, non-overlapping minimum nodes based on the start and end locations of the fragments. For each node, all possible combinations of total copy number (total_cn), allele A copy number (A_cn), and allele B copy number (B_cn) were recorded. We then calculated the detection count for each node and the detection frequency of unique ASCNV fragments characterized by specific combinations of total_cn, A_cn, and B_cn.

Nodes detected at least three times and in a minimum of two analytical processes were retained. Given that each node’s results stemmed from multiple analytical processes, resulting in potential ASCN variation, nodes with an ASCN frequency exceeding 50% were designated as the definitive ASCN for that node.

Finally, adjacent nodes with identical copy numbers were merged, and segments smaller than 3 Mb were removed after merging.

#### Definition of HRD reference datasets

Based on the high-confidence ASCNV fragments identified, the reference datasets were defined to include several key components. The first component consisted of high-confidence ASCNV regions and states across the whole genome, delineated in five columns: chrom, start, end, A_status, and B_status. Here, allele A or B copy number less than or equal to 0.5 was considered a loss, and that greater than or equal to 1.5 was considered a gain. The second component included ASCNV regions for LOH, LST, and TAI. These were analyzed using standardized scarHRD, further annotating segments related to HRD, including LOH, LST, and TAI positions. The dataset comprised four columns: chrom, start, end, and classification. The third component was HRD scores, where high-confidence ASCNVs were analyzed using scarHRD to generate GISs, along with LOH, LST, and TAI scores, which served as reference scores for evaluating HRD.

### Orthogonal validation of ASCNV reference datasets

Each sample generated three ASCNV callsets derived from WGS data, utilizing ASCAT, Facets, and Sequenza for detection. To assess the overlap among these callsets, the multiinter command from BEDTools was employed [51]. This approach identified sub-intervals overlapped by one to three of the callsets, with the numeric count indicating the number of callers that detected each interval.

Intervals with copy number status aligned with those in the reference datasets were selectively retained. The degree of concordance for ASCNV calls was quantified by calculating the total length of intervals detected by any combination of one, two, or all three callers. This length was then compared to the total length of ASCNVs in the reference dataset for each sample, providing a relative measure of concordance across the different detection methodologies.

### Bland-Altman analysis

The Bland-Altman analysis was employed to assess the concordance between HRD scores derived from WGS and panels, in comparison to the reference scores. This analysis entailed computing the mean difference between the two comparative methods, along with its standard deviation (SD). The LoAs were defined as the mean difference ± 1.96 SD. A high level of agreement between methods was inferred when the majority of differences fell within the 95% LoAs, suggesting their potential interchangeability [52,53].

In addition, a one-sample t-test was conducted to determine whether the mean difference significantly deviates from zero. A 95% CI for the mean difference that does not encompass zero indicates a systematic disparity between the two methods. Conversely, if zero falls within the 95% CI of the mean difference, it suggests comparable average results between the two methods. The BlandAltmanLeh package (v0.3.1) in R was utilized to perform these analyses.

### Performance metrics

#### Accuracy of ASCNVs

Precision is the proportion of segments in the test dataset that match the ASCNV status defined in the reference dataset. Recall is the proportion of segments in the reference dataset that are identified in the test dataset with the same ASCNV status. F1 score is the harmonic mean of precision and recall.

#### Reproducibility of ASCNVs

Jaccard index was used to assess the reproducibility of two callsets. This index is defined as the ratio of the intersection length of ASCNVs with matching status to the union length of the two callsets.

#### Accuracy of HRD scores

To quantitatively measure the concordance between panel-derived HRD scores and reference scores, both Pearson correlation coefficients and Bland-Altman analysis were utilized. Percent agreement analysis was employed to assess the consistency of HRD-positive and negative classifications with the reference set at various GIS thresholds.

#### Reproducibility of HRD scores

The CV is defined as the ratio of SD to the mean for every cell line provided by each HRD panel assay.

### Statistical analysis

All statistical analyses were performed using the R statistical software (v4.0.5, https://www.r-project.org).

## Supplementary Material

qzaf017_Supplementary_Data

## Data Availability

The raw sequence data reported in this study have been deposited in the Genome Sequence Archive for Human [54] at the National Genomics Data Center (NGDC), China National Center for Bioinformation (CNCB) (GSA-Human: HRA006776), and are publicly accessible at https://ngdc.cncb.ac.cn/gsa-human/browse/HRA006776.

## CRediT author statement

**Zheng Jia:** Data curation, Formal analysis. **Yaqing Liu:** Formal analysis, Visualization, Validation, Writing – original draft, Writing – review & editing. **Shoufang Qu:** Data curation, Formal analysis. **Wenbin Li:** Conceptualization. **Lin Gao:** Formal analysis, Visualization, Validation, Writing – review & editing. **Lin Dong:** Conceptualization. **Yun Xing:** Data curation, Formal analysis. **Yadi Cheng:** Data curation, Formal analysis. **Huan Fang:** Data curation, Formal analysis. **Yuting Yi:** Data curation, Formal analysis. **Yuxing Chu:** Data curation, Formal analysis. **Chao Zhang:** Data curation, Formal analysis. **Yanming Xie:** Formal analysis, Visualization, Validation. **Zhipeng Xu:** Software, Project administration. **Chunli Wang:** Data curation, Formal analysis. **Zhe Li:** Data curation, Formal analysis. **Zhihong Zhang:** Data curation, Formal analysis. **Yang Wang:** Data curation. **Wenxin Zhang:** Data curation. **Xiaoping Gu:** Data curation, Formal analysis. **Shuang Yang:** Data curation, Formal analysis. **Jinghua Li:** Resources. **Liangshen Wei:** Resources. **Yuanting Zheng:** Formal analysis, Visualization, Validation. **Guohui Ding:** Software, Project administration. **Leming Shi:** Conceptualization, Writing – review & editing, Supervision. **Xin Yi:** Conceptualization, Supervision. **Jianming Ying:** Conceptualization, Formal analysis, Writing – review & editing. **Jie Huang:** Conceptualization, Writing – review & editing. All authors have read and approved the final manuscript.

## ORCID

0009-0009-3665-9193 (Zheng Jia)

0000-0001-9356-9164 (Yaqing Liu)

0000-0002-7202-7160 (Shoufang Qu)

0000-0003-0828-379X (Wenbin Li)

0000-0003-4606-8994 (Lin Gao)

0000-0002-1196-2446 (Lin Dong)

0009-0001-6523-0398 (Yun Xing)

0000-0002-4170-6400 (Yadi Cheng)

0000-0003-1306-1358 (Huan Fang)

0000-0002-3025-4349 (Yuting Yi)

0000-0001-9331-5914 (Yuxing Chu)

0000-0001-9855-7226 (Chao Zhang)

0009-0009-5398-1360 (Yanming Xie)

0009-0008-2729-5618 (Chunli Wang)

0000-0002-5780-5614 (Zhe Li)

0000-0002-1454-1604 (Zhihong Zhang)

0000-0002-0447-9122 (Zhipeng Xu)

0000-0002-4872-0723 (Yang Wang)

0000-0002-7598-9015 (Wenxin Zhang)

0000-0001-6831-9251 (Xiaoping Gu)

0009-0003-9061-0813 (Shuang Yang)

0009-0005-3079-5400 (Jinghua Li)

0009-0003-5632-0935 (Liangshen Wei)

0000-0003-4480-8303 (Yuanting Zheng)

0000-0002-2888-1335 (Guohui Ding)

0000-0002-2981-4150 (Leming Shi)

0000-0003-4032-0298 (Xin Yi)

0000-0002-7301-4118 (Jianming Ying)

0000-0003-2096-8556 (Jie Huang)
