# Supplementary material for: Evaluative Methodology for HRD Testing: Development of Standard Tools for Consistency Assessment
Source: Genomics Proteomics Bioinformatics. 2025 Feb 27;23(1):qzaf017. doi: 10.1093/gpbjnl/qzaf017 (PMC12212637; doi:10.1093/gpbjnl/qzaf017)
Supplement: qzaf017_Supplementary_Data [file qzaf017_supplementary_data.zip › supplementary_material_captions.docx]

# Supplementary material

**Figure S1 Concordance of CNV detection using WES, WGS, and panels**

The median Jaccard Index of ASCN, CN, ASCNS, CNS, detected region (Detect) of the whole-genome regions, as well as the HRD-related regions (LOH, LST, and TAI).

**Figure S2 Concordance of CNV detection of ten cell lines**

The median Jaccard Index for each of the 10 cell lines for ASCN, CN, ASCN, CN, detected region (Detect) of the whole-genome regions, as well as the HRD-related regions (LOH, LST, and TAI).

**Figure S3 Concordance of ASCNS, LOH, LST, TAI of different cell lines**

Heatmaps showcase the concordance of ASCNS, LOH, LST, TAI across cell lines 02, 03, 04, 06, 07, 08, and 09, utilizing three different platforms, nine pipelines across six mass ratios. The black area marks instances where the target variant was undetected in one or both samples within the pairwise comparisons.

**Figure S4** **Ploidy inconsistencies between pipelines reduces ASCNV consistency**

Scatter plots of the CV of ploidy and the Jaccard Index between six pipelines. *R* denotes the Spearman's rank correlation coefficient. Data points represent one sample in each cell line that meet the mass ratio requirements for constructing reference datasets. The solid lines indicate fitted lines obtained from linear regression and the shading indicates the 95% confidence intervals.

**Figure S5 The purity of hyperploidy genomes falls below the theoretical mass ratio**

Scatter plots of the ploidy and the deviation in purity to mass ratio. *R* denotes the Spearman's rank correlation coefficient. Data points represent the median ploidy and median deviation of samples in each cell line that meet the mass ratio requirements for constructing reference datasets. The solid lines indicate fitted lines obtained from linear regression and the shading indicates the 95% confidence intervals.

**Figure S6 HRD scores of WGS data from different pipelines across mass ratios**

Violin plots of GIS, LOH, LST, TAI scores derived from WGS datasets using ASCAT, Facets, and Sequenza.

**Figure S7 Concordance of ASCNV detected by WGS with reference datasets**

Stacked bar plots of the proportion of concordant regions between the reference datasets and WGS-derived ASCNV segments called by ASCAT, Facets, and Sequenza.

**Figure S8** **Reproducibility of ASCNV detection**

Bar plots of Jaccard Index for measuring the reproducibility of detecting ASCNVs for samples that met mass ratio requirements. The number of data instances (*n*) used to derive statistics was as follows: AmD, *n* = 36; BGI, *n* = 36; BnR, *n* = 27; PrS, *n* = 36. Data are presented as mean values ± s.d. The *P* values were calculated using ANOVA tests with false discovery rate (FDR) correction. *****P* < 0.0001, ****P* < 0.001, ***P* < 0.01, **P* < 0.05; not significant, *P* ≥ 0.05.

**Figure S9 Accuracy of ASCNV detection**

Bar plots of F1 scores for measuring the accuracy of detecting ASCNVs for samples that met mass ratio requirements. The number of data instances (*n*) used to derive statistics was as follows: AmD, *n* = 36; BGI, *n* = 36; BnR, *n* = 27; PrS, *n* = 36. Data are presented as mean values ± s.d. The *P* values were calculated using ANOVA tests with false discovery rate (FDR) correction. *****P* < 0.0001, ****P* < 0.001, ***P* < 0.01, **P* < 0.05; not significant, *P* ≥ 0.05.

**Figure S10 Precision and recall for detecting ASCNVs**

Scatter plots of precision and recall of four HRD panels for detecting the ASCNV status, LOH, LST, TAI regions within the high-confidence regions. Samples from all mass ratios are shown (AmD, *n* = 60; BGI, *n* = 60; BnR, *n* = 51; PrS, *n* = 60).

**Figure S11 No significant difference in accuracy assessment metrics**

Bar plots of F1 scores, precision, and recall for measuring the accuracy of detecting ASCNVs. The number of data instances (*n*) used to derive statistics was as follows: AmD, *n* = 36; BGI, *n* = 36; BnR, *n* = 27; PrS, *n* = 36. Data are presented as mean values ± s.d. The *P* values were calculated using ANOVA tests with false discovery rate (FDR) correction. *****P* < 0.0001, ****P* < 0.001, ***P* < 0.01, **P* < 0.05; not significant, *P* ≥ 0.05.

**Figure S12 Accuracy of GIS, LOH, LST, and TAI scores**

Scatter plots of the reference HRD scores and panel-derived HRD scores, including GIS (A), LOH (B), LST (C), and TAI (D). *R* denotes the Spearman's rank correlation coefficient. Data points represent individual samples, with solid lines indicating linear regression fits and shaded areas representing 95% confidence intervals. Samples from all mass ratios are shown (AmD, *n* = 60; BGI, *n* = 60; BnR, *n* = 51; PrS, *n* = 60), but only samples meeting quality requirements were included in statistical analyses (AmD, *n* = 36; BGI, *n* = 36; BnR, *n* = 27; PrS, *n* = 36).

**Figure S13** **Association of high HRD score bias with low ASCNV accuracy**

Scatter plots of the absolute bias of reference HRD scores and the F1 scores of ASCNV segments. *R* denotes the Spearman's rank correlation coefficient. The number of data instances (*n*) used to derive statistics was as follows: AmD, *n* = 36; BGI, *n* = 36; BnR, *n* = 27; PrS, *n* = 36. Data points represent one sample and solid lines indicate fitted lines obtained from linear regression. The shading indicates the 95% confidence intervals.

**Table S1 Genomic coverage of WES, GM-seq, and WGS**

**Table S2 Mutations associated with defective BRCA function**

**Table S3 Potential differentially methylated HRR genes**

**Table S4 Blacklisted genomic regions for constructing reference datasets**
