# Supplementary material for: Evaluative Methodology for HRD Testing: Development of Standard Tools for Consistency Assessment
Source: Genomics Proteomics Bioinformatics. 2025 Feb 27;23(1):qzaf017. doi: 10.1093/gpbjnl/qzaf017 (PMC12212637; doi:10.1093/gpbjnl/qzaf017)
Supplement: qzaf017_Supplementary_Data [file qzaf017_supplementary_data.zip › Table_S1.docx]

**Table S1 Genome coverage of WES, GM-seq, and WGS**

| **Cell line** | **WES** | **GM-seq** | **WGS** |
| --- | --- | --- | --- |
| 01 | 546.07 | 113.63 | 147.86 |
| 02 | 672.71 | 111.42 | 177.10 |
| 03 | 612.21 | 106.08 | 190.77 |
| 04 | 680.64 | 137.92 | 181.49 |
| 05 | 578.57 | 125.89 | 143.83 |
| 06 | 697.21 | 122.07 | 190.11 |
| 07 | 773.29 | 121.43 | 190.81 |
| 08 | 521.43 | 114.40 | 152.69 |
| 09 | 739.64 | 133.09 | 163.78 |
| 10 | 725.29 | 126.31 | 178.88 |

*Note*: Coverage (X) was represented as the average depth of deduplicated mapped reads of targeted capture regions for WES and the whole genome regions for GM-seq and WGS. The coverage of WES and WGS in the table is the average of 14 datasets per cell line, and GM-seq corresponds to two datasets.
