# Supplementary material for: Evaluative Methodology for HRD Testing: Development of Standard Tools for Consistency Assessment
Source: Genomics Proteomics Bioinformatics. 2025 Feb 27;23(1):qzaf017. doi: 10.1093/gpbjnl/qzaf017 (PMC12212637; doi:10.1093/gpbjnl/qzaf017)
Supplement: qzaf017_Supplementary_Data [file qzaf017_supplementary_data.zip › Table_S3.docx]

**Table S3 Potential differentially methylated HRR genes**

| **Gene** | **Cell line** | **Tumor AvgFreqT** | **Normal AvgFreqT** | **logFC** | **Class** |
| --- | --- | --- | --- | --- | --- |
| *BRCA1* | 01 | 11.66 | 70.25 | -2.51 | down |
| *BRCA1* | 05 | 14.61 | 73.26 | -2.26 | down |
| *BRCA2* | 10 | 16.75 | 44.11 | -1.36 | down |
| *EID3* | 02 | 10.66 | 46.28 | -2.06 | down |
| *ERCC1* | 08 | 6.75 | 31.51 | -2.16 | down |
| *LIG1* | 06 | 5.21 | 36.35 | -2.71 | down |
| *PARG* | 05 | 0.00 | 46.80 | -6.64 | down |
| *RAD51B* | 02 | 9.08 | 63.17 | -2.70 | down |
| *RAD54B* | 02 | 16.30 | 43.65 | -1.38 | down |
| *RBBP8* | 04 | 13.21 | 27.20 | -1.01 | down |
| *SHFM1* | 02 | 18.02 | 44.30 | -1.26 | down |
| *SLX4* | 06 | 0.00 | 24.30 | -6.64 | down |
| *SLX4* | 07 | 7.40 | 21.02 | -1.47 | down |
| *SLX4* | 08 | 2.46 | 25.20 | -3.22 | down |
| *SMC5* | 05 | 4.69 | 22.59 | -2.20 | down |
| *SWSAP1* | 06 | 10.26 | 40.86 | -1.94 | down |
| *TP53BP1* | 06 | 6.97 | 21.89 | -1.61 | down |
| *XRCC2* | 06 | 14.45 | 37.28 | -1.33 | down |
| *EID3* | 03 | 89.34 | 44.32 | 1.02 | up |
| *EID3* | 07 | 89.12 | 42.22 | 1.08 | up |
| *RPA3* | 02 | 81.50 | 35.13 | 1.22 | up |

*Note*: Average frequency of thymidine (AvgFreqT) indicates the methylation level in the gene promoter region, with log2 fold change (logFC) representing the difference between tumor and normal samples. To prevent infinite values, 0.01 was added to the fold change before calculating the log2 ratio. Class designates whether the methylation level is upregulated or downregulated in tumor samples compared to normal samples. Down is defined when tumor AvgFreqT is less than 20%, normal AvgFreqT exceeds 20%, and logFC is below 1. Up is defined when tumor AvgFreqT exceeds 80%, normal AvgFreqT is below 80%, and logFC is above 1. HRR, homologous recombination repair
