# Supplementary material for: Evaluative Methodology for HRD Testing: Development of Standard Tools for Consistency Assessment
Source: Genomics Proteomics Bioinformatics. 2025 Feb 27;23(1):qzaf017. doi: 10.1093/gpbjnl/qzaf017 (PMC12212637; doi:10.1093/gpbjnl/qzaf017)
Supplement: qzaf017_Supplementary_Data [file qzaf017_supplementary_data.zip › Table_S4.docx]

**Table S4 Blacklisted genomic regions for constructing reference datasets**

| **Cell line** | **Chr** | **Start** | **End** | **Label** |
| --- | --- | --- | --- | --- |
| 01 | 11 | 128,447,762 | 134,944,141 | sparseSNP |
| 01 | 15 | 796,429,60 | 102,396,557 | ctrlCNV |
| 04 | 6 | 122,629,901 | 154,864,124 | ctrlCNV |
| 04 | 16 | 465,840,97 | 901,243,15 | sparseSNP |
| 05 | 8 | 0 | 146,364,022 | ctrlCNV |
| 05 | 12 | 0 | 133,851,895 | ctrlCNV |
| 05 | 13 | 661,047,89 | 115,086,043 | ctrlCNV |
| 06 | 6 | 148,039 | 275,645,47 | ctrlCNV |
| 06 | 13 | 739,260,85 | 115,086,043 | ctrlCNV |
| 06 | 15 | 0 | 102,531,392 | ctrlCNV |
| 09 | 6 | 204,908 | 587,606,83 | sparseSNP |
| 09 | 16 | 465,840,97 | 901,243,15 | sparseSNP |

*Note*: Label is the reason for labeling the region as blacklisted, sparseSNP stands for low SNP density, and ctrlCNV stands for CNV detection in the region of the control sample. SNP, single nucleotide polymorphisms; CNV, copy number variation.
