# Supplementary material for: Evaluative Methodology for HRD Testing: Development of Standard Tools for Consistency Assessment
Source: Genomics Proteomics Bioinformatics. 2025 Feb 27;23(1):qzaf017. doi: 10.1093/gpbjnl/qzaf017 (PMC12212637; doi:10.1093/gpbjnl/qzaf017)
Supplement: qzaf017_Supplementary_Data [file qzaf017_supplementary_data.zip › Table_S2.docx]

**Table S2 Mutations associated with defective *BRCA* function**

| **Cell line** | **Origin** | **Defeat gene** | **cHGVS** | **pHGVS** |
| --- | --- | --- | --- | --- |
| 07 | somatic | BRCA1 | c.5425_5426delGT | p.V1809Cfs*20 |
| 09 | somatic | BRCA2 | c.4777G > T | p.E1593* |
| 09 | germline | BRCA1 | c.5251C > T | p.R1751* \| p.Arg1751* |
| 10 | germline | BRCA1 | c.5266dupC | p.Q1756Pfs*74 \| p.Gln1756Profs*74 |
