# Supplementary figures and images for: Evaluative Methodology for HRD Testing: Development of Standard Tools for Consistency Assessment
Source: Genomics Proteomics Bioinformatics. 2025 Feb 27;23(1):qzaf017. doi: 10.1093/gpbjnl/qzaf017 (PMC12212637; doi:10.1093/gpbjnl/qzaf017)

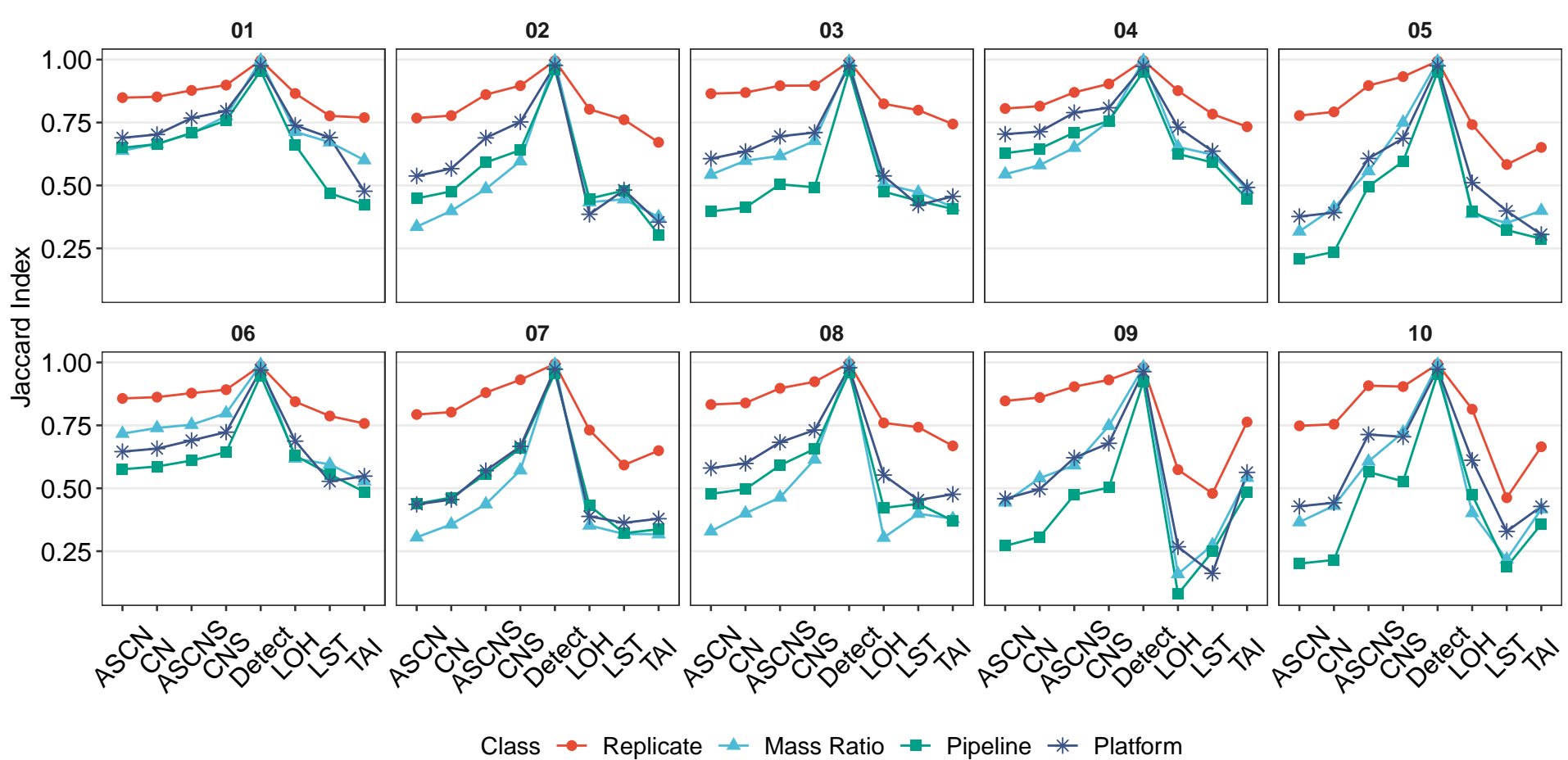

Supplement: qzaf017_Supplementary_Data [file qzaf017_supplementary_data.zip › Figure_S2.pdf]

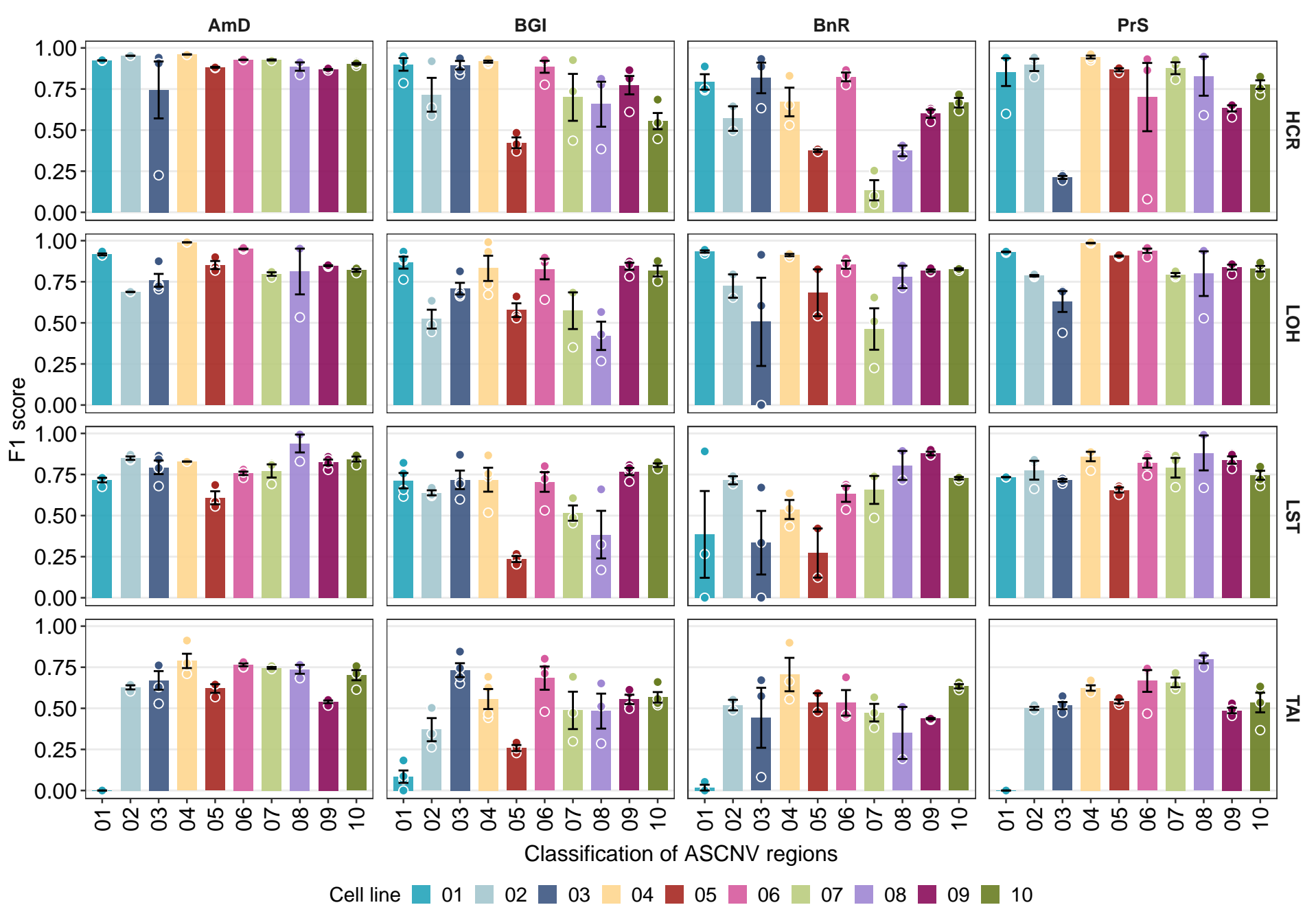

Supplement: qzaf017_Supplementary_Data [file qzaf017_supplementary_data.zip › Figure_S9.pdf]

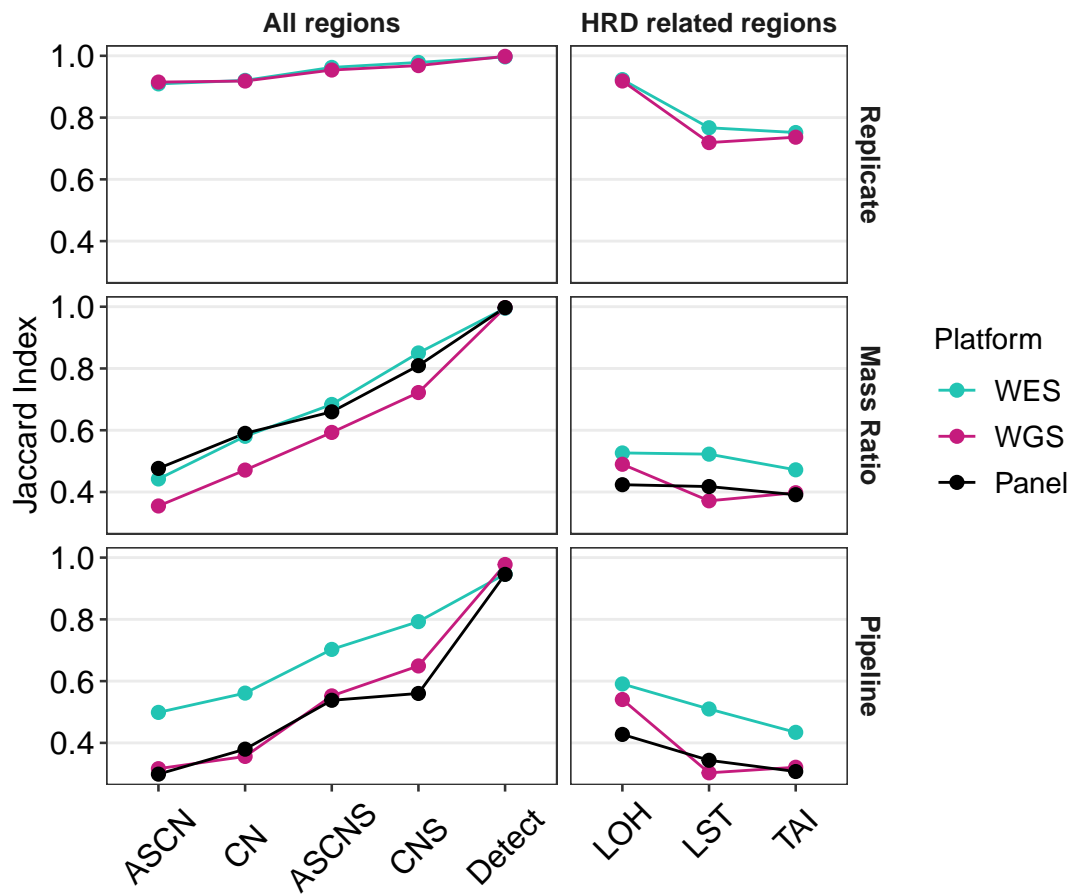

Supplement: qzaf017_Supplementary_Data [file qzaf017_supplementary_data.zip › Figure_S1.pdf]

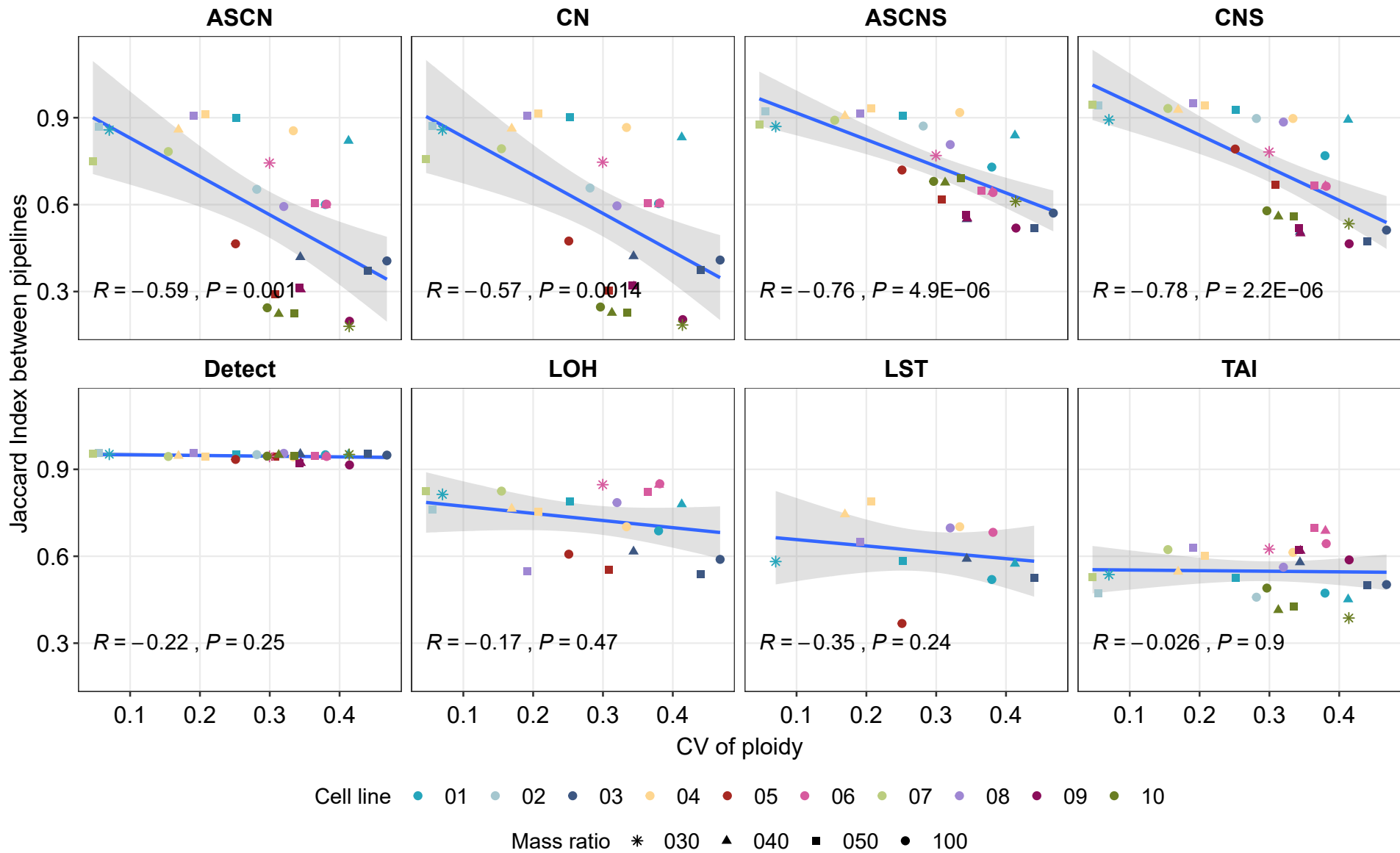

Supplement: qzaf017_Supplementary_Data [file qzaf017_supplementary_data.zip › Figure_S4.pdf]

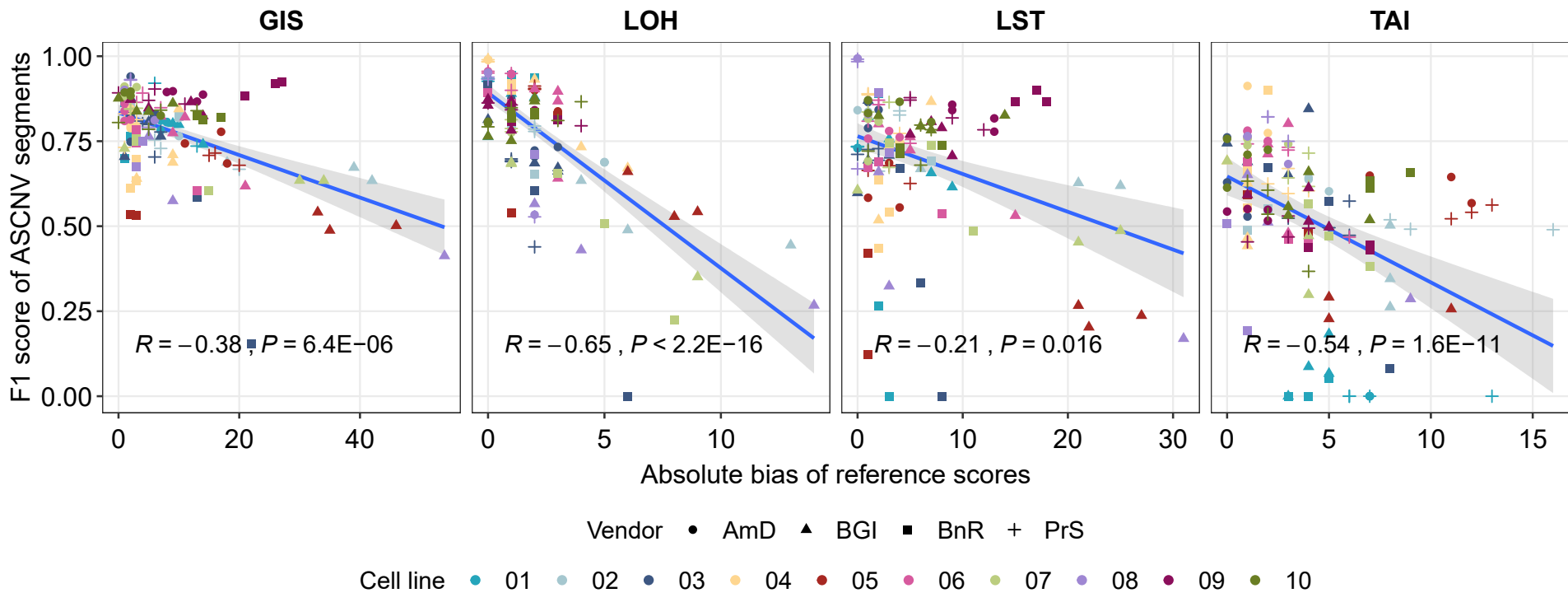

Supplement: qzaf017_Supplementary_Data [file qzaf017_supplementary_data.zip › Figure_S13.pdf]

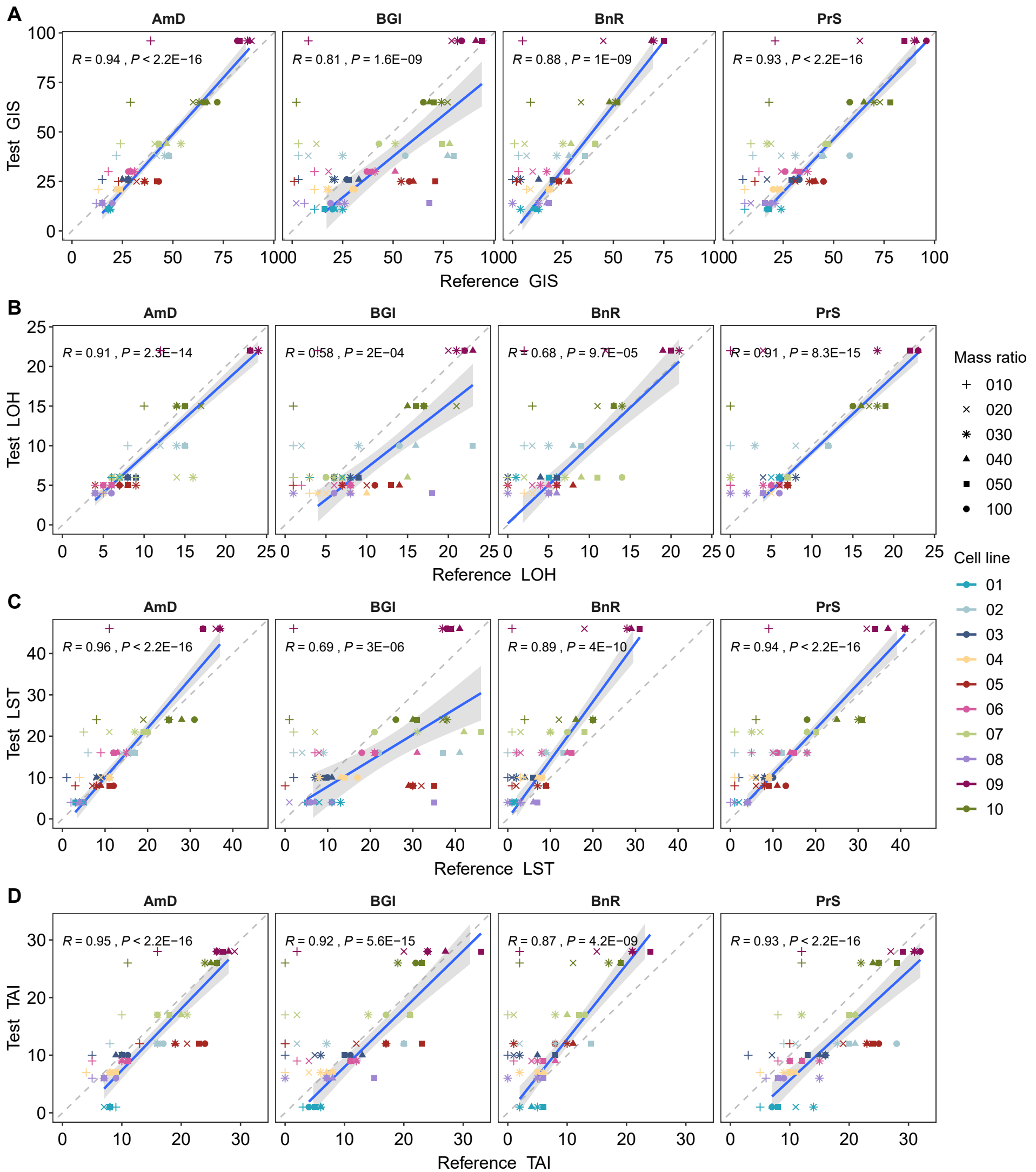

Supplement: qzaf017_Supplementary_Data [file qzaf017_supplementary_data.zip › Figure_S12.pdf]

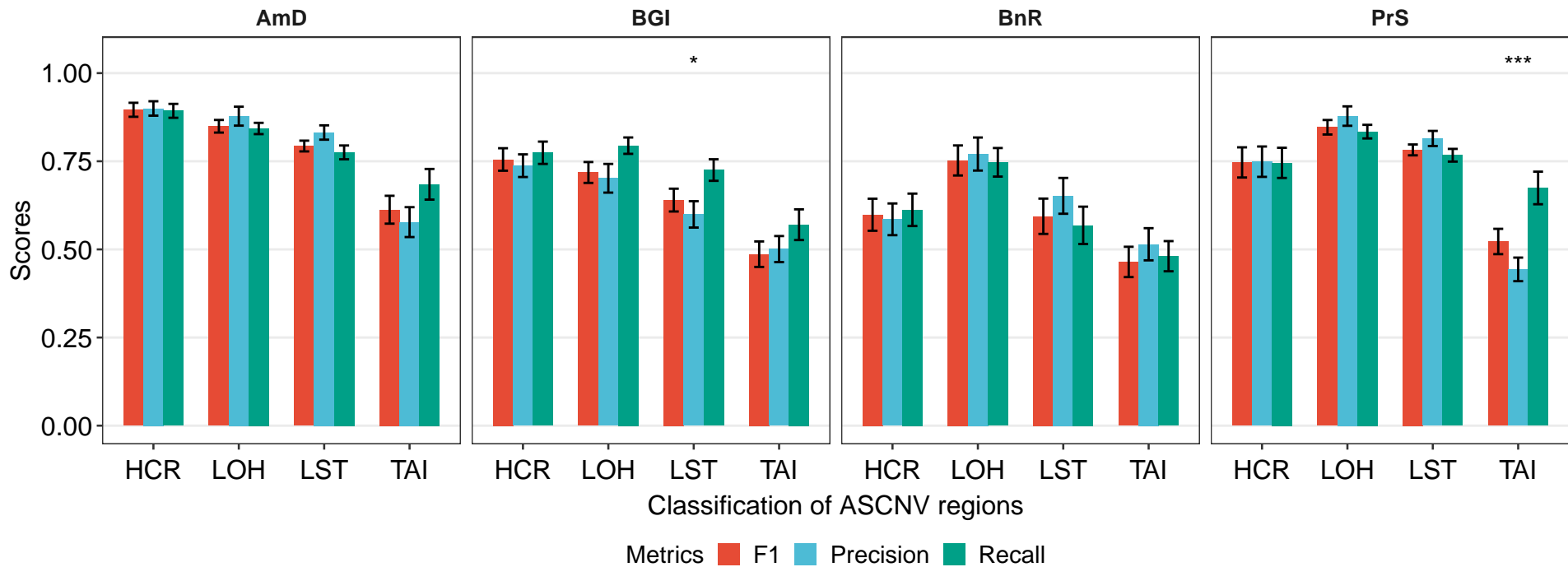

Supplement: qzaf017_Supplementary_Data [file qzaf017_supplementary_data.zip › Figure_S11.pdf]

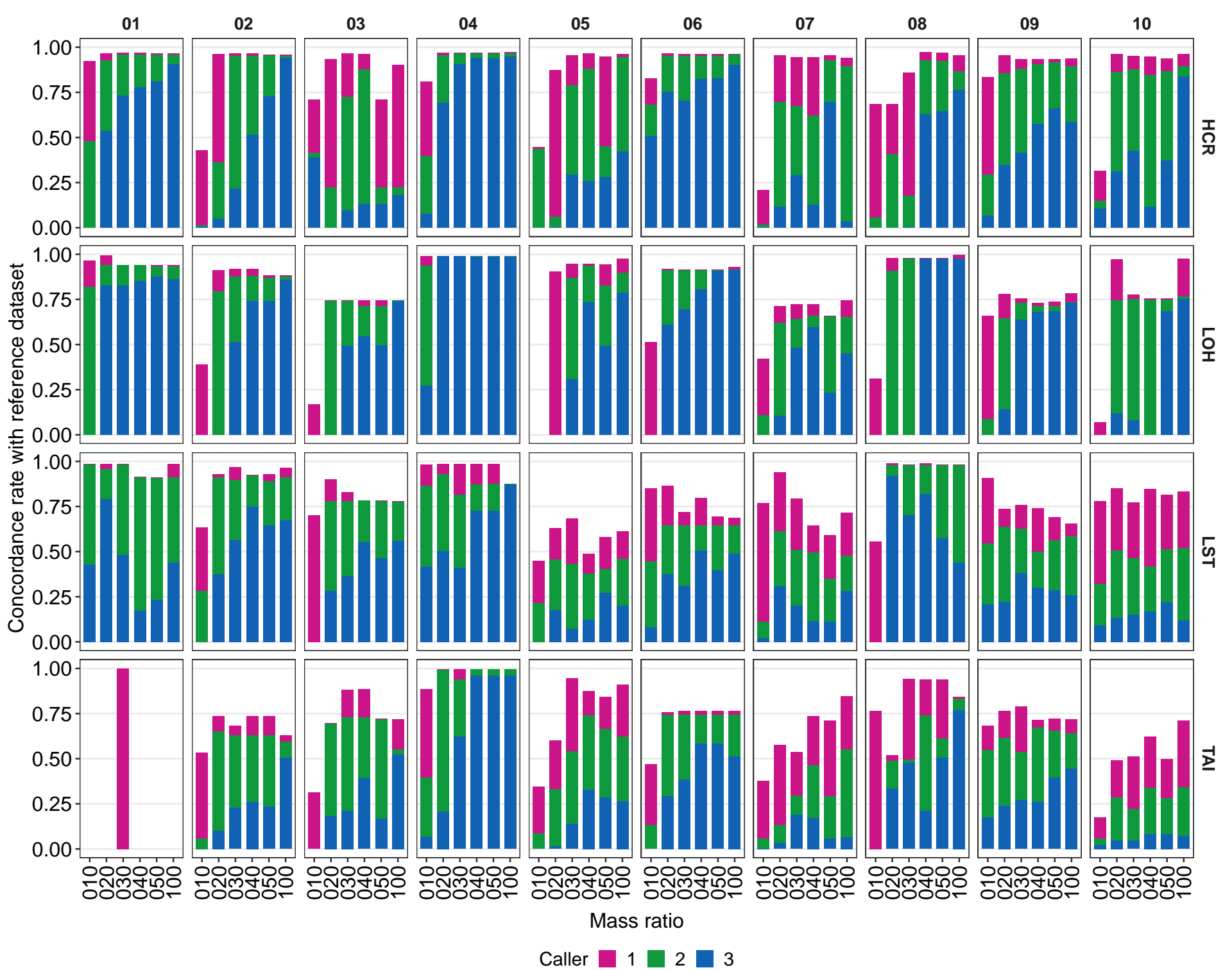

Supplement: qzaf017_Supplementary_Data [file qzaf017_supplementary_data.zip › Figure_S7.pdf]

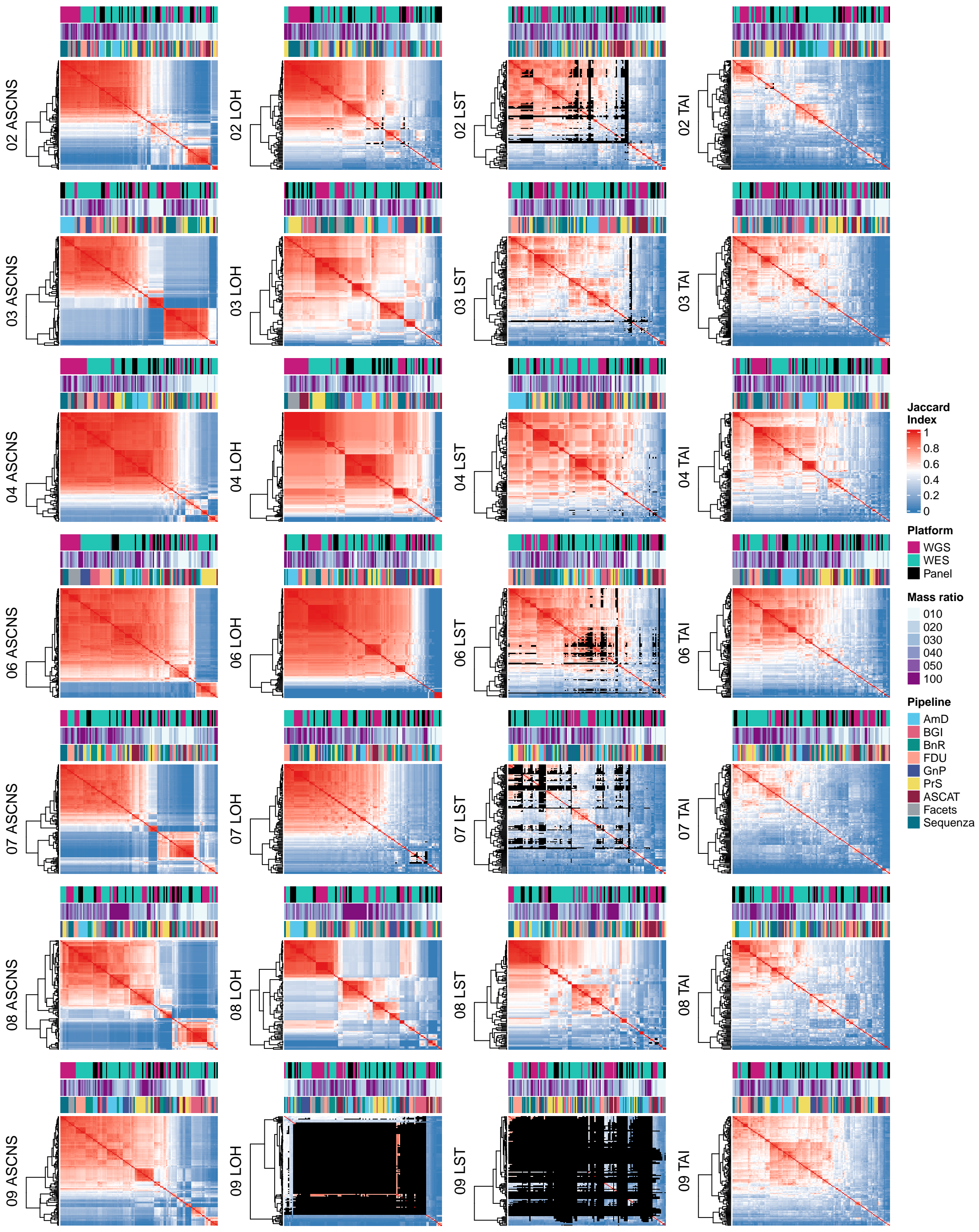

Supplement: qzaf017_Supplementary_Data [file qzaf017_supplementary_data.zip › Figure_S3.pdf]

Deviation in purity to mass ratio

$R = -0.75$ ,  $P < 2.2\text{E-}16$

Cell line

- 01
- 02
- 03
- 04
- 05
- 06
- 07
- 08
- 09
- 10

2

3

4

5

Ploidy

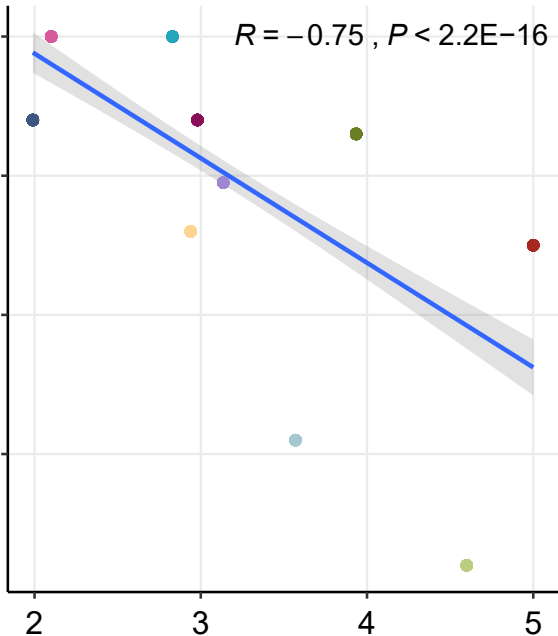

Supplement: qzaf017_Supplementary_Data [file qzaf017_supplementary_data.zip › Figure_S5.pdf]

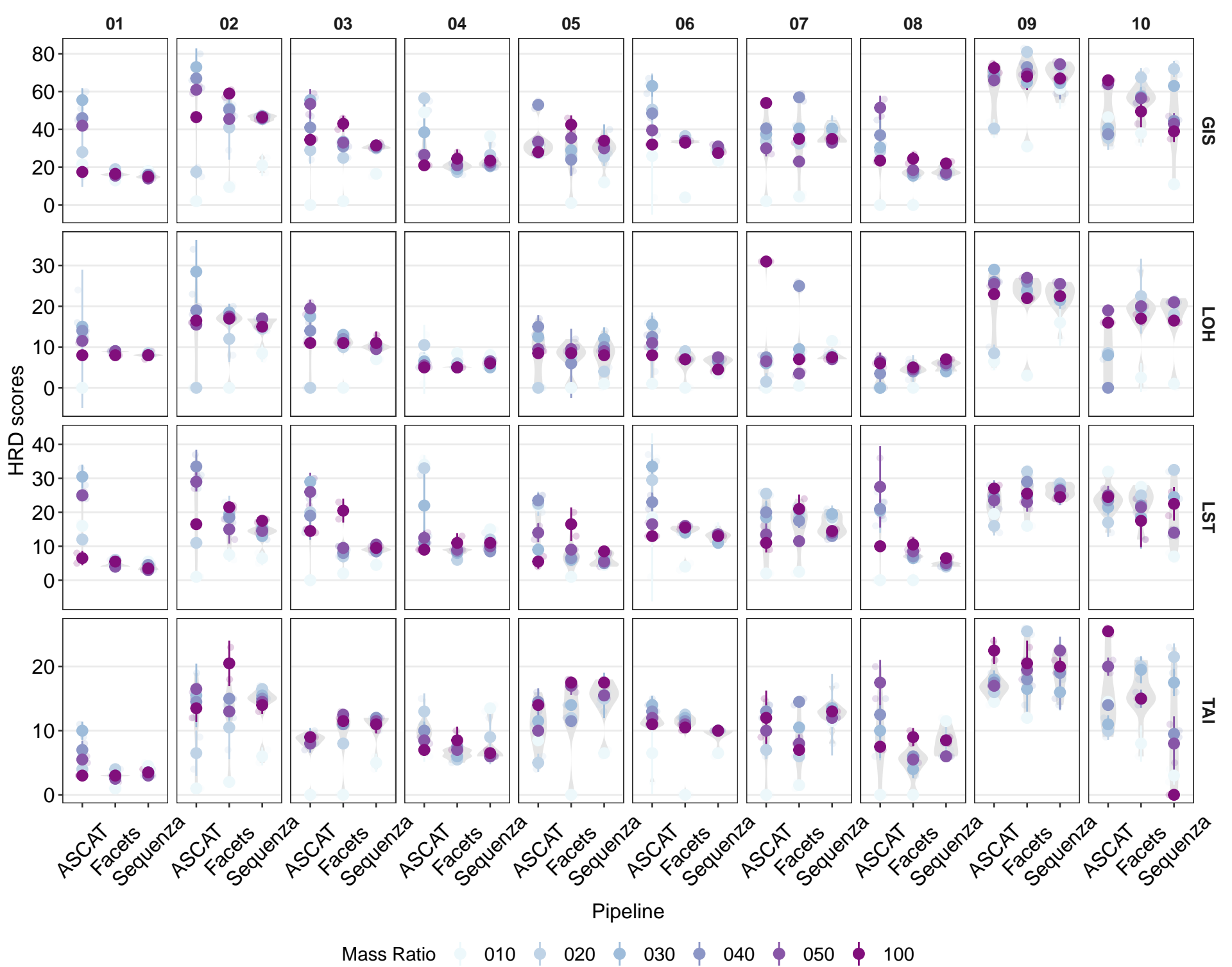

Supplement: qzaf017_Supplementary_Data [file qzaf017_supplementary_data.zip › Figure_S6.pdf]

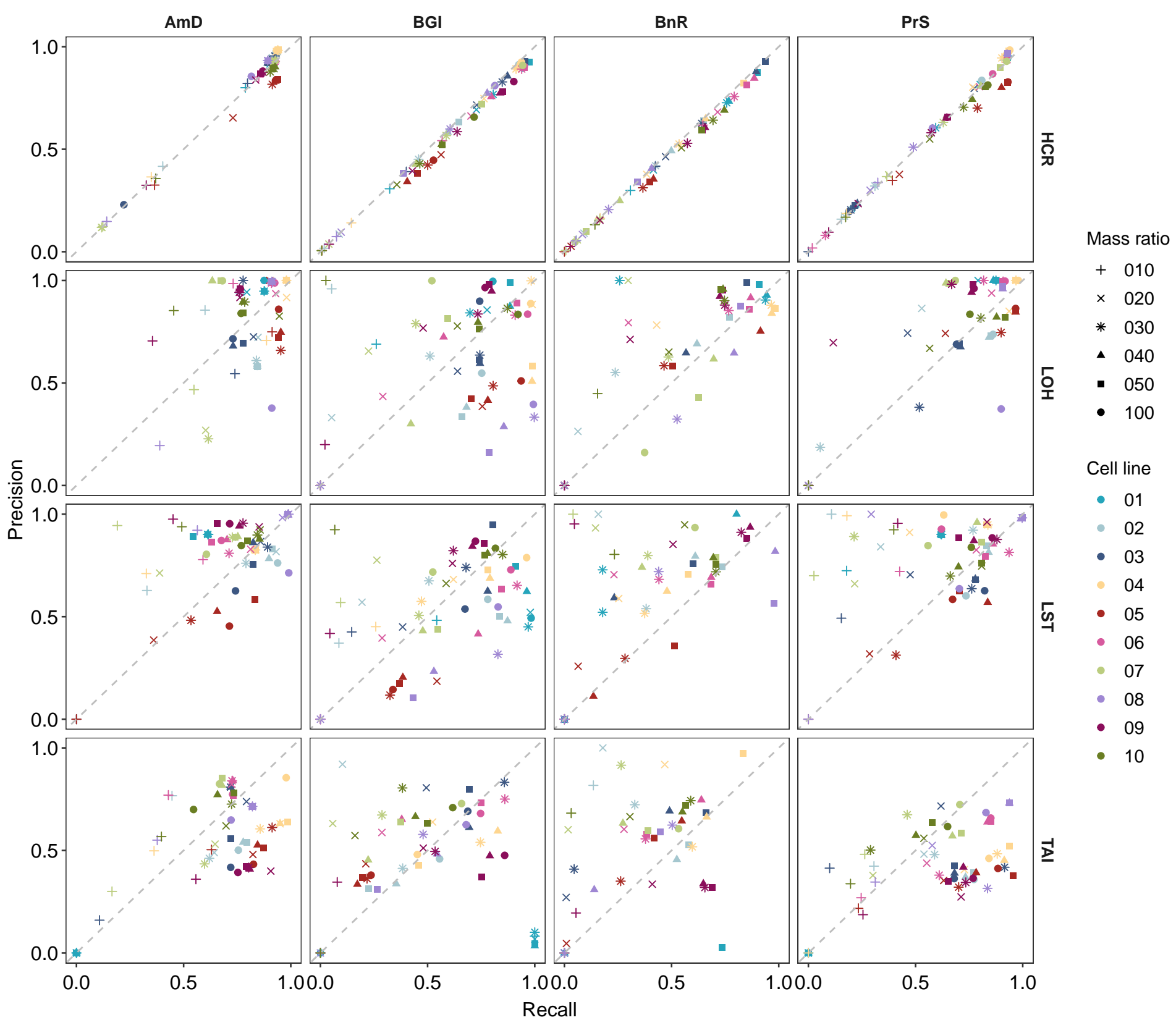

Supplement: qzaf017_Supplementary_Data [file qzaf017_supplementary_data.zip › Figure_S10.pdf]

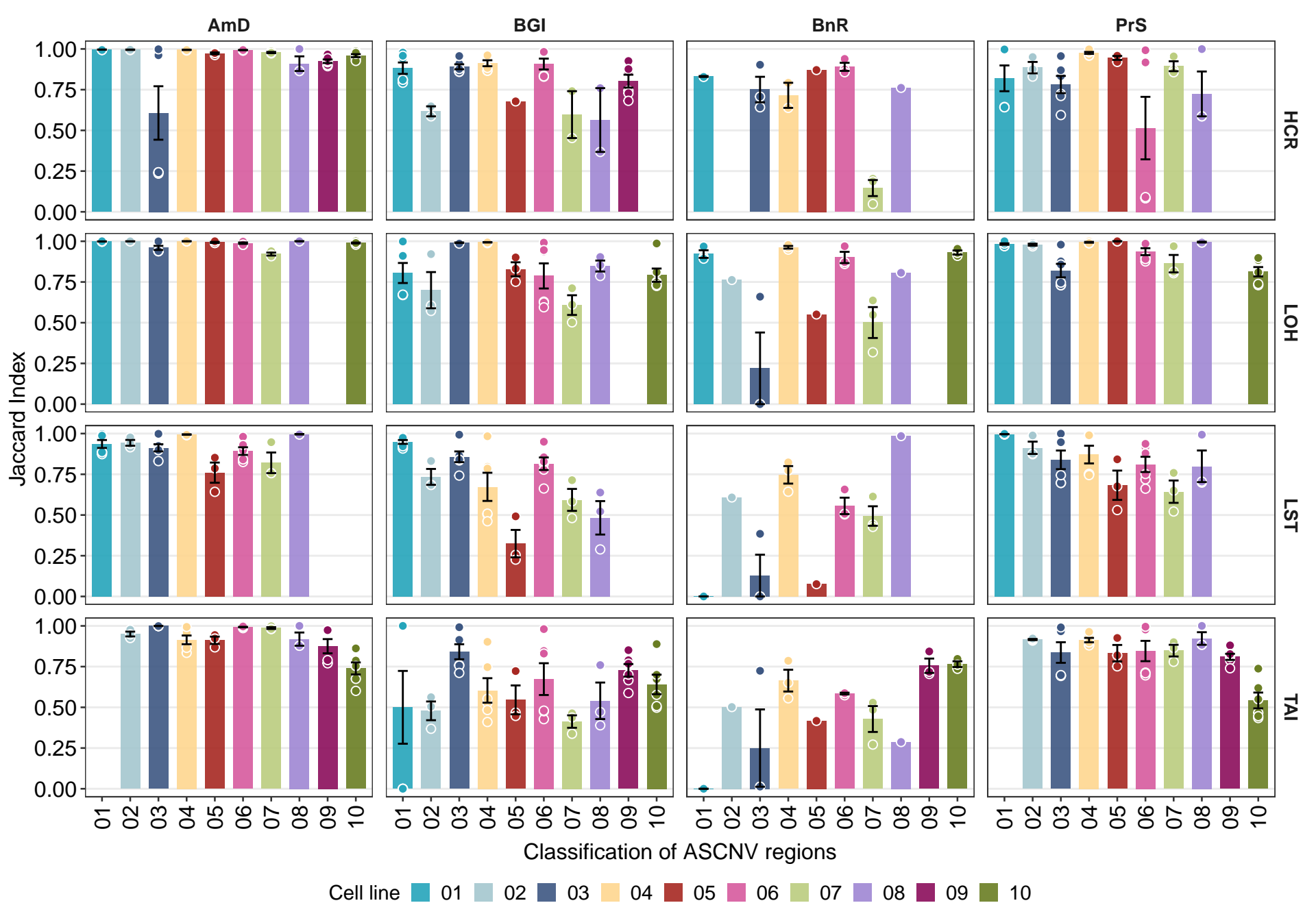

Supplement: qzaf017_Supplementary_Data [file qzaf017_supplementary_data.zip › Figure_S8.pdf]
